# New Lactones Produced by *Streptomyces* sp. SN5431 and Their Antifungal Activity against *Bipolaris maydis*

**DOI:** 10.3390/microorganisms11030616

**Published:** 2023-02-28

**Authors:** Yinan Wang, Di Yang, Zhiguo Yu

**Affiliations:** 1 College of Plant Protection, Shenyang Agricultural University, Shenyang 110866, China; 2 Engineering & Technological Research Center of Biopesticide for Liaoning Province, Shenyang 110866, China

**Keywords:** *Streptomyces*, *γ*-butyrolactones, *Bipolaris maydis*, antifungal activity

## Abstract

*Bipolaris maydis* causes southern corn leaf blight and inflicts huge losses on maize production. In order to search for new natural products from insect gut bacteria to control plant fungal disease, 86 actinomycetes were isolated from more than 50 insect guts, in which crude extract of strain SN5431 showed significant inhibition of the mycelial growth of *B. maydis*. The strain was identified and named as *Streptomyces* sp. SN5431. Six compounds were obtained from the crude extract of strain SN5431, which includes five new *γ*-butyrolactones named as tiuslactone A–E (**1**–**5**), and one new long chain ester named as tiusester (**6**). Their structures were determined using NMR and HRESIMS data and then combined with the spectroscopic data of known similar compounds. Tiuslactone B (**2**) showed powerful antifungal activity against *B. maydis*. These results indicated metabolites of insect gut bacteria have the potential to be the leading compounds for the control of corn leaf blight.

## 1. Introduction

Maize is extensively planted in many countries of the world, such as the United States, China, India, etc. [1,2,3]. In addition to its pivotal role in food, maize is also used as an indispensable raw material in the energy and package industries [4,5]. Therefore, the quality and quantity of maize are positively correlated with the development of national economies [6]. However, the Southern corn leaf blight (SCLB) caused by *Bipolaris maydis* can lead to millions of losses in the production of maize [7]. Traditionally, chemical agents were generally applied to control SCLB, including carbendazim and mancozeb [8,9]. Unfortunately, some stains of *B. maydis* had developed resistance to commercially available fungicides in many regions [9]. In this case, there is an urgent need of finding promising candidate fungicides.

The secondary metabolites of insect intestinal microorganisms can be sources of pesticides or their precursors, which has successfully attracted the attention of many natural product chemists [10,11]. In recent years, a large number of novel biological activity compounds, including diterpenoids, lactones, alkaloids and linear peptides have been reported by scholars. [12,13,14,15]. These unique compounds have played important roles as crop protection agents with the potential to address complex agricultural problems. 

More than half of commercially available antibiotics are produced by *Streptomyces* [16]. *γ*-Butyrolactones regulate the antibiotic production and differentiation of *Streptomyces* [17,18,19]. Among them, A-factor is a typical *γ*-butyrolactone, which presents a hydroxymethyl group at C-2 and a fatty acid residue at C-3 [20,21,22]. In addition, *γ*-butyrolactones are prominent structural elements in natural products due to their broad, biologically active antibacterial, antifungal and anti-inflammatory effects [23,24].

**Figure 2:**
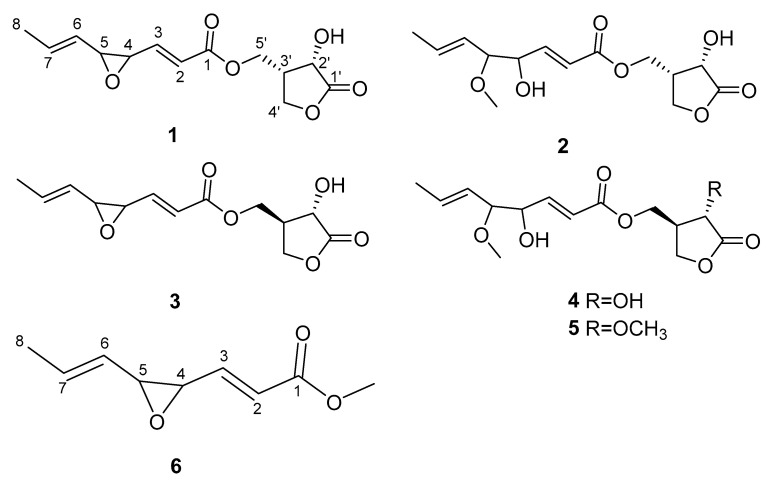
Structures of compounds **1**–**6**.

In this study, strain SN5431 was isolated from the grub gut samples and its secondary metabolite showed strong inhibition of *B. maydis*. Moreover, five new *γ*-butyrolactones (**1**–**5**) (Figure 2) and one new long chain ester (**6**) (Figure 2) were separated from the CH_2_Cl_2_ extract of strain SN5431. The research could provide useful information for controlling SCLB.

## 2. Materials and Methods

### 2.1. General Experimental Procedures

The AP-300 polarimeter (Atago, Tokyo, Japan) was used to obtain optical rotations. NMR spectra were *measured on* an Avance-600 NMR spectrometer (Bruker, Karlsruhe, Germany) at 25 °C. Hydrogen signal and carbon signals of CDCl_3_ (δ_H_ 7.26 and δ_C_ 77.0) were used for calibration. *High-resolution mass spectra* (HRESIMS were recorded on a 6500 series quadrupole time-of-flight (Q-TOF) mass spectrometer (Agilent, Santa Clara, CA, USA). High-performance liquid chromatography (HPLC) analysis was performed on a 1260 Infinity LC system (Agilent, Santa Clara, CA, USA). Semipreparative HPLC was performed on a 1260 series system (Agilent, Santa Clara, CA, USA). Column chromatography was performed using Sephadex LH-20 (GE Healthcare, Uppsala, Sweden) and silica gel (100–200 mesh and 200–300 mesh) (Qingdao Ocean Chemical Co., Ltd., Qingdao, China). All chemical reagents were purchased from a chemical reagent company (Sinopharm Chemical Reagent Co., Ltd., Shanghai, China).

### 2.2. Sample Collection

Insect samples were collected from Shenyang City, Liaoning Province, China. They were placed in different boxes. After bringing the insect samples back to the laboratory, they were placed at room temperature. The insects were identified by the Insect Classification Laboratory, Shenyang Agricultural University.

### 2.3. Isolation of Actinomycetes

The collected insect samples were immersed in 75% alcohol for 5 min to ensure that the insect surface was fully disinfected. They were washed three times in sterile distilled water, after which the surface moisture of the insects was blotted dry with sterile filter paper. Gut dissections were performed using sterile tools (tweezers, scissors and scalpel) and then pulverized in sterile mortars. After adding 1 mL of sterile distilled water in mortars, the supernatant samples were collected and subjected to 10-fold serial dilutions (from 10^−1^ to 10^−6^). Each dilution (100 μL) was plated on Gause’s synthetic agar no. 1 (GS) [25] and supplemented with potassium dichromate solution. After ten days, colonies were transferred and purified on GS for subsequent experiments.

### 2.4. Screening and Identification of Antagonistic Actinobacteria Strains

Isolated strains were inoculated into a 250 mL Erlenmeyer flask containing 50 mL of GS liquid medium and 2 g of Amberlite XAD-16 resin. After seven days of incubation at 180 rpm and 28 °C, the resin was collected and extracted four times with MeOH. The combined MeOH extracts were concentrated under a reduced pressure to obtain crude extracts. They were tested for antifungal activity against *B. maydis* using the mycelial growth inhibition method [26]. Briefly, mycelial discs (5 mm in diameter) were placed on PDA plates amended with 300 μg/mL crude extracts. Plates without crude extracts were considered as the control. After seven days, the diameters of the colonies were taken. The inhibitory rate of the crude extracts on the growth of *B. maydis* was calculated. 

According to the screening results, the active strain was identified by using molecular and phenotypic procedures. The strain was observed for mycelial and spore morphology. For genotypic identification, the 16S rRNA sequence of the strain was compared with the those available in the EzTaxon database. The phylogenetic tree was inferred based on the 16S rRNA sequence using the neighbor-joining algorithm [27]. 

### 2.5. Extraction and Isolation

Crude extract (2.71 g) was obtained from the fermentation culture (43.2 L) of active strain via conventional laboratory methods [28]. It was fractionated via silica gel chromatography (CH_2_Cl_2_-MeOH, 100:0 → 1:1) to give seven fractions (Fr. A–G). Fr. E (382.2 mg) was subjected to Sephadex LH-20 column chromatography (CC) (MeOH-CH_2_Cl_2_, 1:1) to obtain five subfractions (Fr. E1–E5). Fr. E2 (32.5 mg) was further fractionated via silica gel CC (petroleum ether-ethyl acetate, 65:35) to yield three subfractions (Fr. E2A–E2C). Fr. E2B was repeatedly subjected to Sephadex LH-20 CC (petroleum ether–dichloromethane–methanol, 2:1:1) to obtain compound **3** (11.7 mg). Fr. E2C was subjected to Sephadex LH-20 CC (MeOH-CH_2_Cl_2_, 1:1) to give compounds **2** (25.3 mg) and **4** (11.4 mg). Fr. F was subjected to silica gel CC (petroleum ether–ethyl acetate, 65:35) and Sephadex LH-20 CC (petroleum ether–dichloromethane–methanol, 2:1:1) to obtain Fr. F1 and compounds **1** (7.4 mg) and **6** (4.4 mg). Fr. F1 was subsequently separated via reverse-phase semipreparative HPLC (MeOH-H_2_O) to yield compound **5** (6.9 mg, *t*_R_ 58 min, 25% MeOH).

Tiuslactone A, **1**: Pale yellow oil; ${\left[\alpha \right]}_{D}^{24}-21.65$ (c 0.77, MeOH); HRESIMS *m/z* 269.1021 [M + H]^+^ (calcd. for C_13_H_17_O_6_, 269.1025).

Tiuslactone B, **2**: Pale yellow oil; ${\left[\alpha \right]}_{D}^{24}-19.44$ (c 1.20, MeOH); HRESIMS *m/z* 301.1276 [M + H]^+^ (calcd. for C_14_H_21_O_7_, 301.1287).

Tiuslactone C, **3**: Pale yellow oil; ${\left[\alpha \right]}_{D}^{24}-29.13$ (c 1.03, MeOH); HRESIMS *m/z* 269.1022 [M + H]^+^ (calcd. for C_13_H_17_O_6_, 269.1025).

Tiuslactone D, **4**: Pale yellow oil; ${\left[\alpha \right]}_{D}^{24}+11.70$ (c 1.14, MeOH); HRESIMS *m/z* 301.1276 [M + H]^+^ (calcd. for C_14_H_21_O_7_, 301.1287).

Tiuslactone E, **5**: Pale yellow oil; ${\left[\alpha \right]}_{D}^{24}-21.74$ (c 0.46, MeOH); HRESIMS *m/z* 315.1468 [M + H]^+^ (calcd. for C_15_H_23_O_7_, 315.1443).

Tiusester, **6**: Pale yellow oil; ${\left[\alpha \right]}_{D}^{24}+22.73$ (c 0.44, MeOH); HRESIMS *m/z* 169.0859 [M + H]^+^ (calcd. for C_9_H_13_O_3_, 169.0864).

### 2.6. Microdilution Broth Assay 

The antifungal activity of compounds **1**–**6** against *B. maydis* was evaluated using the microdilution broth [29]. For the microdilution broth assay, mycelia were taken from *B. maydis* incubated for 7 days and placed on a centrifuge tube with a 1.5 mL RPMI-1640 medium and two steel balls. They then were ground for 8 min to obtain fungal suspension. All tested compounds (100 μL) and fungal suspension (100 μL) were added to the 96-well plate to reach a final concentrations agent concentration of 100 µg/mL. Chlorothalonil (CHT) was used as a positive control; the same volume of dimethyl sulfoxide was considered as the negative control. After 48 h, the optical density was measured. Experiments were repeated three times. The percentage of the growth inhibition was calculated using the following formula:

$$\mathrm{inhibition}\;(\%)=(1-\;\frac{\mathrm{OD}\;\mathrm{of}\;\mathrm{treated}\;\mathrm{well}\;}{\mathrm{OD}\;\mathrm{of}\;\mathrm{negative}\;\mathrm{control}\;\mathrm{well}\;})\;\times \;100$$

### 2.7. Mycelial Growth Inhibition Assay

The mycelial growth inhibition assay was performed via the previously described method [26]. The PDA medium with 0.5% DMSO was considered as the negative control. CHT was used as the positive control. 

## 3. Results

### 3.1. Isolation and Screening of Strain SN5431

A total of 86 actinomycetes were isolated from more than 50 insect guts and then named as strain SN5402–SN5487. They were stored in the Laboratory of Microbial Metabolites, College of Plant Protection, at Shenyang Agricultural University, Shenyang, China.

The crude extracts of the isolated actinomycetes showed different levels of inhibition activity against *B. maydis*, in which the crude extracts of strain SN5431 could completely inhibit the mycelial growth of *B. maydis* at 300 μg/mL. Therefore, we chose strain SN5431 as a candidate strain. 

### 3.2. Identification of Strain SN5431

**Figure 1:**
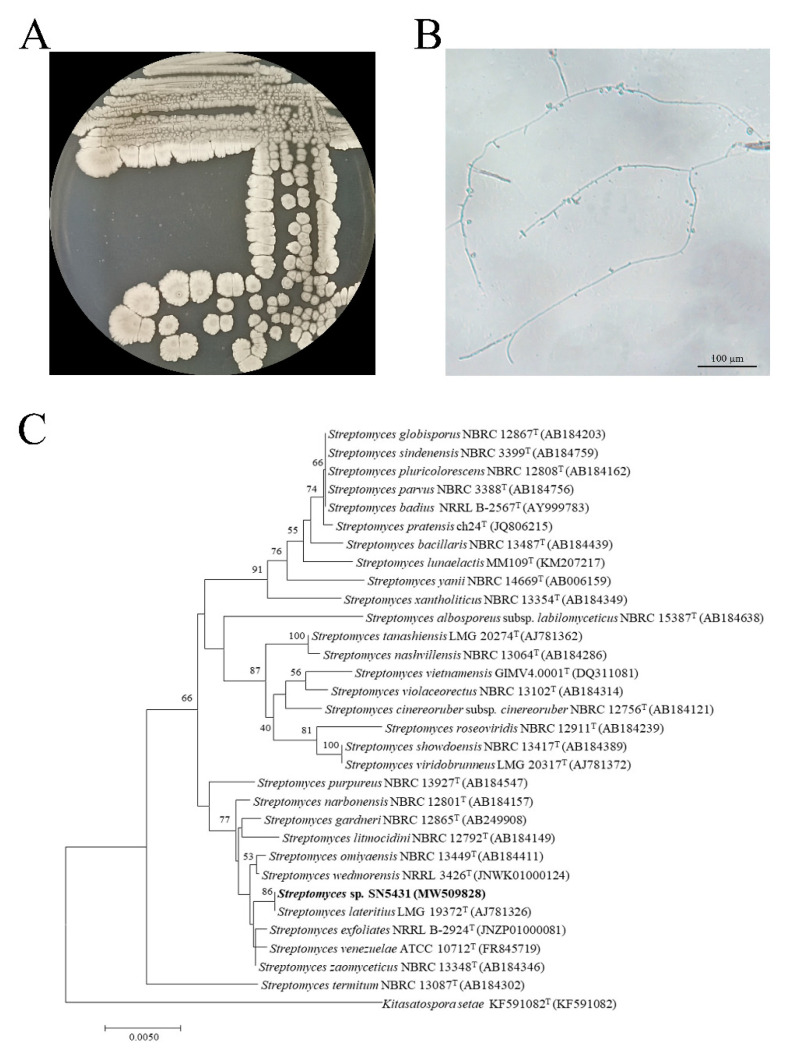
The identification of strain SN5431. (**A**) The colony morphology of strain SN5431 on GS medium. (**B**) The morphology of strain SN5431 on GS medium. (**C**) Neighbor-joining phylogenetic tree based on the 16S rRNA gene sequences with strain SN5431 and related members of the genus *Streptomyces*.

Strain SN5431, isolated from the intestine of *Larva Holotrichiae*, was identified via phylogenetic analysis and compared to 16S rRNA gene sequences in EzTaxon database. The sequence of the 16S rRNA has the greatest similarity to *Streptomyces lateritius* (99.3%). The phylogenetic analysis showed the stain can form a cluster with *Streptomyces lateritius* LMG 19372^T^ (Figure 1C). The morphology of strain SN5431 on GS was typical of *Streptomyces* with a white mycelia (Figure 1A,B). Thus, strain SN5431 belongs to the genus *Streptomyces* (Genbank accession no. MW509828).

### 3.3. Structure Elucidation of Compounds 

**Figure 3:**
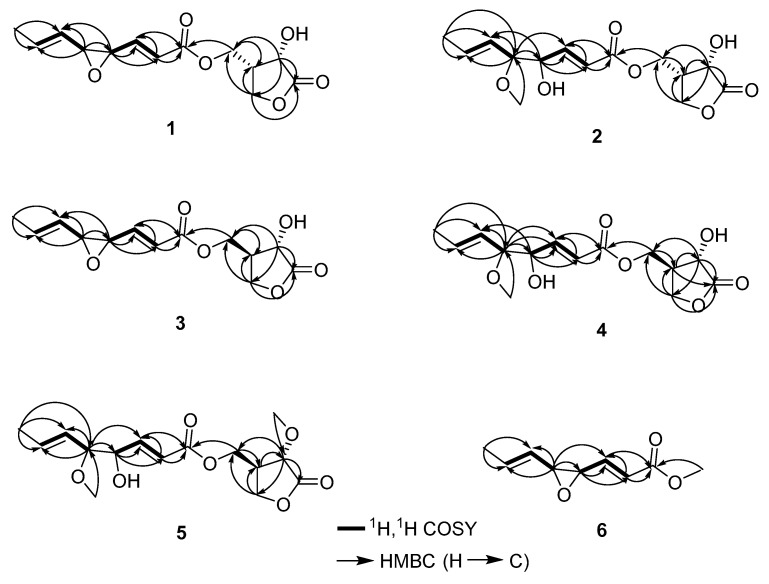
Key COSY (bold) and HMBC (arrows) correlations of compounds **1**–**6**.

Compound **1** was isolated as a pale-yellow oil. The molecular formula, C_13_H_16_O_6_, was established via the positive HRESIMS ion peak at *m/z* 269.1021 [M + H]^+^ (Figure S7). The ^1^H NMR data of **1** (Table 1 and Figure S1) showed one methyl proton signal [*δ*_H_ 1.75 (dd, 3H, H_3_-8)], four olefinic proton signals [*δ*_H_ 5.22 (m, 1H, H-6), *δ*_H_ 5.98 (dq, 1H, H-7), *δ*_H_ 6.08 (dd, 1H, H-2) and *δ*_H_ 6.71 (dd, 1H, H-3)] and three oxygenated methine groups [*δ*_H_ 3.28 (m, 1H, H-5), *δ*_H_ 3.35 (dd, 1H, H-4), *δ*_H_ 4.58 (d, 1H, H-2′)]. The ^13^C NMR spectrum of **1** (Table 2 and Figure S2) showed 13 carbon signals attributed to two ester carbonyl groups, eight methines (including three oxygenated methines), two methylenes and one methyl. The COSY correlations of H-2′/H-3′/H-4′ and the HMBC correlations from H-2′, H-3′ and H-4′ to C-1′ unambiguously illustrated the presence of the *γ*-lactone ring (Figure 3, Figures S4 and S5). The unsaturated epoxy ester unit was deduced according to COSY and HMBC correlations. The COSY correlations illustrated the connectivity of H-2/H-3/H-4/H-5/H-6/H-7/H-8. The HMBC correlations from H-5′ (*δ*_H_ 4.35), H-2 (*δ*_H_ 6.08) and H-3 (*δ*_H_ 6.71) to C-1 (*δ*_C_ 165.3) illustrated the positions of one ester carbonyl group. The above structure unit was linked at C-3′ (*δ*_C_ 38.9), which was determined by the HMBC correlations from H-5′ to C-3′. Further, the coupling constants of ^3^*J*_2H–3H_ = 15.7 Hz and ^3^*J*_6H–7H_ = 15.4 Hz indicated H-2/H-3 and H-6/H-7 are *trans* orientations. The NOESY cross-peak between H-2′ and H-3′ suggested that H-2′ and H-3′ were cofacial (Figure S6). Additionally, the coupling constant of H-2′ for compound **1** are consistent with that of H-2 of lactone Ⅱ [30,31,32], which indicated that the relative configurations of C-2′ and C-3′ in **1** are 2′*S* and 3′*S*. Therefore, compound **1** was elucidated and named as tiuslactone A (Figure 2).

Compound **2** was obtained as pale-yellow oil. The molecular formula C_14_H_20_O_7_ was established via the positive HRESIMS ion peak at *m/z* 301.1276 [M + H]^+^ (Figure S14). The analysis of their data showed a structural similarity between compounds **2** and **1** as *γ*-lactone derivative (Figures S8–S13). The comparison of the NMR data of **2** with those of **1** showed that there were obvious differences in the unsaturated epoxy ester part, in which chemical shifts of C-4 (*δ*_C_ 72.8) and C-5 (*δ*_C_ 84.6) were significantly shifted downfield in **2** (Table 2). The ^1^H and ^13^C NMR data revealed the presence of the methoxy group and hydroxyl group in **2** rather than the epoxy group in **1**, which was supported by the HRESIMS data (Table 1 and Table 2). The methoxy group was linked at C-5, based on the HMBC correlations from the methoxy protons (*δ*_H_ 3.29) to C-5. From these results, **2** was elucidated and named as tiuslactone B (Figure 2).

Compound **3** was isolated as a pale-yellow oil. Its molecular formula C_13_H_16_O_6_ was established via the HRESIMS data (Figure S20), same as the situation in 1. Analysis of the NMR data also indicated that the structure of **3** was similar to that of **1** (Figures S15–S19). The striking NMR differences between them are the chemical shifts of H-2′, H-3′ and H-4′ owing to the different configuration of C-3′, which agrees well with reported musacins B_1_ in the literature [32]. Therefore, **3** was elucidated and named as tiuslactone C (Figure 2). 

Compound **4** was isolated as a pale-yellow oil and had the same molecular formula as **2**, C_14_H_20_O_7_, compatible with their HRESIMS data (Figure S27). Further analysis of their NMR data indicated that they possessed similar 2D structures (Figures S21–S26). Notably, the deshielding of C-2′ (*δ*_C_ 68.8, Δ*δ*_C_ + 1.1) and C-3′ (*δ*_C_ 43.3, Δ*δ*_C_ + 4.3) in **4** suggested that C-3′was of an *R* configuration. The NOESY correlation of H-2′/H-3′ verified the abovementioned changes (Figure S26). Consequently, **4** was identified and named as tiuslactone D (Figure 2). 

Compound **5** was purified as a pale-yellow oil with a molecular formula of C_15_H_22_O_7_ compatibly with its HRESIMS data (Figure S34). Its NMR data was remarkably similar to that of **4** (Figures S28–S33). In detail, the ^1^H and ^13^C NMR data of **5** showed an additional methoxy group instead of the hydroxyl group in **4**, agreeing with a 14 Da mass decrease. Further, the HMBC correlation of H_3_-2′OCH_3_ with C-2′ revealed that the methoxy group (*δ*_H_ 3.77, *δ*_C_ 52.8) was connected to C-2′ (Figure 3). As a result, **5** was elucidated and named as tiuslactone E (Figure 2).

Compound **6** was isolated as pale-yellow oil and possessed the molecular formula C_9_H_12_O_3_ via HRESIMS analysis (Figure S41). Comparing the ^1^H and ^13^C NMR spectra of **6** with **1**, all signals could be easily recognized (Table 1 and Table 2), except for the *γ*-lactone ring at C-1′ to C-4′ that have absented in **6**. Therefore, 6 had no *γ*-lactone skeleton and it was a long-chain ester. The structure of **6** was determined and **6** was named as tiusester (Figure 2).

### 3.4. Antifungal Activity Assay

**Figure 4:**
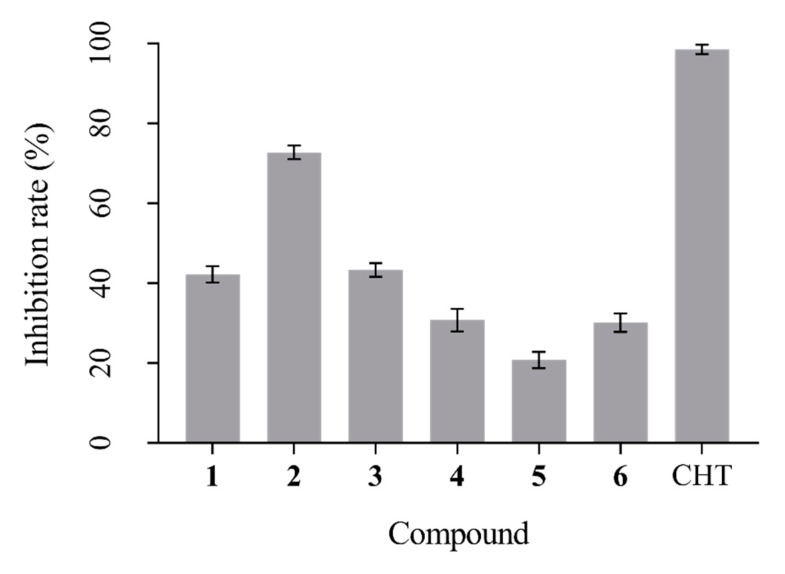
The inhibitory effect of tested compounds on *B. maydis* at 100 μg/mL with chlorothalonil (CHT) as positive control.

In the microdilution broth assay, isolated compounds’ preliminarily antifungal activities were shown in Figure 4. Compound **2** exhibited a strong inhibitory effect on *B. maydis*, and its inhibition rate was 73.23% at 100 ug/mL. Further, other compounds had weaker inhibitory activity than compound **2** (Figure 4). 

In the mycelial growth inhibition assay, compound **2** also showed a good inhibitory effect on *B. maydis*. However, other compounds had no antifungal activity against *B. maydis* (Table 3).

## 4. Discussion

Maize is the staple food in different parts of the world, and it is extensively planted [33]. However, it’s growers are facing the tricky problems caused by pests, such as pathogens, insects and weeds. Among these pests, SCLB caused a 10–46% yield loss in epidemic years [34]. So far, SCLB is still a serious disease due to racial variations [35]. Therefore, the development of new pesticides is urgent and necessary. Previous studies indicated natural products were studied to control disease [36,37]. Actinomyces can produce multiple bioactive secondary metabolites. In this study, 86 actinomycetes were isolated to screen the candidate for controlling the SCLB. The candidate actinomycete was shown to belong to the Streptomyces genus via phylogenetic analysis and was named *Streptomyces* sp. SN5431. The crude extract (300 μg/mL) can completely inhibit the mycelial radical elongation of *B. maydis*.

Six new compounds **1**–**6** were isolated and purified from the cultures of *Streptomyces* sp. SN5431, among which tiuslactones A–E (**1**–**5**) are *γ*-butyrolactones and tiusester (**6**) is a long-chain ester. Compound **6** seems to be an artefact, produced by the methanol-induced hydrolyses of compound **1**. The relative configuration of these compounds was deduced by comparing their NMR data with those of similar compounds [31], but their absolute configuration could not be deduced from the existing information. During the experiment, we used different methods to culture the crystals of isolated compounds; however, we did not succeed due to the structural characteristics of compounds. 

It is widely known that biological control plays an important role in agricultural industry due to its ecological safety and potential to fulfil sustainable corn development. However, the information of actinomyces based on biological compounds against *B. maydis* is limited. In this study, compound **2**, produced by *S. lateritius* SN5431, has an obvious inhibitory effect on *B. maydis*. Therefore, the biologically active compound has the ability to become the leading molecule for controlling agricultural diseases.

## 5. Conclusions

In conclusion, *S. lateritius* SN5431 was isolated from the intestine of *Larva Holotrichiae*. Further, the crude extract from strain SN5431 showed inhibitory activity against *B. maydis*. Compounds **1**–**6** were obtained from the crude extract, among which compounds **1**–**5** were new *γ*-butyrolactones and **6** was a new long-chain ester. The structures of **1**–**6** have been determined using the NMR and HRESIMS data and combined with the spectroscopic data of known similar compounds (Supplementary Materials). Compound **2** showed good antifungal activity against *B. maydis*. However, its actual efficacy in the field needs to be further studied.

## Figures and Tables

**Table 1:** ^1^H (600 MHz) NMR data of compounds **1**–**6** in CDCl_3_.

Position	*δ*_H_, Mult (*J* in Hz)					
	1	2	3	4	5	6
1-OCH_3_						3.75 s (1.3)
2	6.08 d (15.6)	6.09 dd (15.7, 1.9)	6.13 dd (15.7, 0.7)	6.12 dd (15.7, 1.9)	6.10 dd (15.7, 1.8)	6.13 dd (16.2, 1.8)
3	6.71 dd (15.7, 6.7)	6.96 dd (15.7, 4.3)	6.78 dd (15.7, 6.6)	7.00 dd (15.7, 0.7)	6.97 dd (15.7, 4.4)	6.96 dd (15.8, 4.6)
4	3.35 dd (6.6, 1.4)	4.29 overlap	3.37 m	4.33 overlap	4.33 overlap	4.34 dd (4.4, 1.7)
5	3.28 m	3.66 m	3.28 dd (8.6, 1.8)	3.65 dd (8.4, 4.6)	3.65 m	4.19 m
5-OCH_3_		3.29 s		3.28 s	3.29 s	
6	5.22 m	5.35 m	5.23 m	5.33 m	5.34 m	5.51 m
7	5.98 dq (15.4, 6.6)	5.80 m	5.99 dq (15.4, 6.5)	5.79 m	5.79 m	5.82 m
8	1.75 dd (6.7, 1.6)	1.76 dd (6.5, 1.5)	1.76 dd (6.6, 1.6)	1.76 dd (6.5, 1.6)	1.76 dd (6.5, 1.4)	1.74 m
2′	4.58 d (8.0)	4.57 d (8.0)	4.34 overlap	4.33 overlap	4.33 overlap	
2′-OCH_3_					3.81 s	
3′	2.98 m	2.98 d (1.7)	2.87 m	2.86 m	2.45 m	
4′α	4.35 overlap	4.29 overlap	4.44 overlap	4.05 t (9.8)	4.33 overlap	
4′β	4.45 dd (11.6, 3.9)	4.47 dd (11.6, 4.0)	4.04 dd (10.1, 9.6)	4.04 dd (10.1, 9.6)	3.76 dd (13.2, 6.6)	
5′α	4.35 overlap	4.37 m	4.44 overlap	4.33 overlap	4.33 overlap	
5′β	4.35 overlap	4.37 m	4.34 overlap	4.41 dd (8.6, 1.8)	4.33 overlap	

**Table 2:** ^13^C (150 MHz) NMR data of compounds **1**−**6** in CDCl_3_.

Position	*δ*_C_, Type					
	1	2	3	4	5	6
1	165.3 qC	166.0 qC	165.3 qC	166.0 qC	166.4 qC	166.7 qC
1-OCH_3_						51.7 CH_3_
2	122.2 CH	120.8 CH	122.1 CH	120.6 CH	121.3 CH	122.1 CH
3	145.7 CH	147.5 CH	145.8 CH	147.7 CH	146.9 CH	145.7 CH
4	57.8 CH	72.8 CH_2_	57.7 CH	72.8 CH_2_	72.9 CH_2_	73.7 CH
5	61.4 CH	84.6 CH	61.5 CH	84.6 CH	84.6 CH	75.2 CH
5-OCH_3_		56.4 CH_3_		56.3 CH_3_	56.3 CH_3_	
6	127.1 CH	126.4 CH	127.0 CH	126.4 CH	126.5 CH	128.3 CH
7	133.0 CH	132.9 CH	133.2 CH	133.0 CH	132.9 CH	130.7 CH
8	17.9 CH_3_	17.9 CH_3_	17.9 CH_3_	17.9 CH_3_	17.9 CH_3_	17.9 CH_3_
1′	176.5 qC	176.4 qC	176.4 qC	176.5 qC	174.8 qC	
2′	67.5 CH	67.7 CH	68.9 CH	68.8 CH	69.9 CH	
2′-OCH_3_					52.8 CH_3_	
3′	38.9 CH	39.0 CH	43.2 CH	43.3 CH	43.5 CH	
4′	61.0 CH_2_	60.8 CH_2_	66.8 CH_2_	66.8 CH_2_	60.0 CH_2_	
5′	67.8 CH_2_	67.8 CH_2_	61.7 CH_2_	61.5 CH_2_	62.2 CH_2_	

**Table 3:** Half maximal effective concentration (EC_50_) values of compounds **1**–**6** on *B. maydis*.

Compound	EC_50_, µg/mL (±SD)
Tiuslactone A, **1**	>150
Tiuslactone B, **2**	95.31 ± 2.09
Tiuslactone C, **3**	>150
Tiuslactone D, **4**	>150
Tiuslactone E, **5**	>150
Tiusester, **6**	>150
Chlorothalonil ^a^	3.20 ± 0.19

^a^ Positive control.

## Data Availability

Not applicable.

## Supplementary Materials

The following supporting information can be downloaded at: https://www.mdpi.com/article/10.3390/microorganisms11030616/s1, Figures S1–S41: HRESI-MS and NMR spectra of compounds **1**–**6**.
