# Supplementary material for: New Lactones Produced by Streptomyces sp. SN5431 and Their Antifungal Activity against Bipolaris maydis
Source: Microorganisms. 2023 Feb 28;11(3):616. doi: 10.3390/microorganisms11030616 (PMC10056170; doi:10.3390/microorganisms11030616)

Support information

New Lactones Produced by *Streptomyces* sp. SN5431 and Their Antifungal Activity against *Bipolaris maydis*

Yinan Wang [1], Di Yang [1] and Zhiguo Yu [1,2,*]

[1] College of Plant Protection, Shenyang Agricultural University, Shenyang 110866, China
[2] Engineering & Technological Research Center of Biopesticide for Liaoning Province, Shenyang 110866, China
* Correspondence: zyu@syau.edu.cn; Tel.: +86-24-88487148

Contents

Figure S1. The ^{1}H-NMR spectrum of Tiuslactone A, **1**, in $CDCL_3$ 1

Figure S2. The ^{13}C-NMR spectrum of Tiuslactone A, **1**, in $CDCL_3$ 1

Figure S3. The HSQC spectrum of Tiuslactone A, **1**, in $CDCL_3$ 2

Figure S4. The HMBC spectrum of Tiuslactone A, **1**, in $CDCL_3$ 2

Figure S5. The ^{1}H-^{1}H COSY spectrum of Tiuslactone A, **1**, in $CDCL_3$ 3

Figure S6. The NOESY spectrum of Tiuslactone A, **1**, in $CDCL_3$ 3

Figure S7. The HRESIMS spectrum of Tiuslactone A, **1** 4

Figure S8. The ^{1}H-NMR spectrum of Tiuslactone B, **2**, in $CDCL_3$ 4

Figure S9. The ^{13}C-NMR spectrum of Tiuslactone B, **2**, in $CDCL_3$ 5

Figure S10. The HSQC spectrum of Tiuslactone B, **2**, in $CDCL_3$ 5

Figure S11. The HMBC spectrum of Tiuslactone B, **2**, in $CDCL_3$ 6

Figure S12. The ^{1}H-^{1}H COSY spectrum of Tiuslactone B, **2**, in $CDCL_3$ 6

Figure S13. The NOESY spectrum of Tiuslactone B, **2**, in $CDCL_3$ 7

Figure S14. The HRESIMS spectrum of Tiuslactone B, **2** 7

Figure S15. The ^{1}H-NMR spectrum of Tiuslactone C, **3**, in $CDCL_3$ 8

Figure S16. The ^{13}C-NMR spectrum of Tiuslactone C, **3**, in $CDCL_3$ 8

Figure S17. The HSQC spectrum of Tiuslactone C, **3**, in $CDCL_3$ 9

Figure S18. The HMBC spectrum of Tiuslactone C, **3**, in $CDCL_3$ 9

Figure S19. The ^{1}H-^{1}H COSY spectrum of Tiuslactone C, **3**, in $CDCL_3$ 10

Figure S20. The HRESIMS spectrum of Tiuslactone C, **3** 10

Figure S21. The ^{1}H-NMR spectrum of Tiuslactone D, **4**, in $CDCL_3$ 11
Figure S22. The ^{13}C-NMR spectrum of Tiuslactone D, **4**, in $CDCL_3$..................................... 11
Figure S23. The HSQC spectrum of Tiuslactone D, **4**, in $CDCL_3$ 12
Figure S24. The HMBC spectrum of Tiuslactone D, **4**, in $CDCL_3$... 12
Figure S25. The ^{1}H-^{1}H COSY spectrum of Tiuslactone D, **4**, in $CDCL_3$.............................. 13
Figure S26. The NOESY spectrum of Tiuslactone D, **4**, in $CDCL_3$ 13
Figure S27. The HRESIMS spectrum of Tiuslactone D, **4**... 14
Figure S28. The ^{1}H-NMR spectrum of Tiuslactone E, **5**, in $CDCL_3$ 14
Figure S29. The ^{13}C-NMR spectrum of Tiuslactone E, **5**, in $CDCL_3$ 15
Figure S30. The HSQC spectrum of Tiuslactone E, **5**, in $CDCL_3$ 15
Figure S31. The HMBC spectrum of Tiuslactone E, **5**, in $CDCL_3$ 16
Figure S32. The ^{1}H-^{1}H COSY spectrum of Tiuslactone E, **5**, in $CDCL_3$ 16
Figure S33. The NOESY spectrum of Tiuslactone E, **5**, in $CDCL_3$.. 17
Figure S34. The HRESIMS spectrum of Tiuslactone E, **5**... 17
Figure S35. The ^{1}H-NMR spectrum of Tiuslactone F, **6**, in $CDCL_3$ 18
Figure S36. The ^{13}C-NMR spectrum of Tiuslactone F, **6**, in $CDCL_3$ 18
Figure S37. The HSQC spectrum of Tiuslactone F, **6**, in $CDCL_3$ 19
Figure S38. The HMBC spectrum of Tiuslactone F, **6**, in $CDCL_3$ 19
Figure S39. The ^{1}H-^{1}H COSY spectrum of Tiuslactone F, **6**, in $CDCL_3$................................ 20
Figure S40. The NOESY spectrum of Tiuslactone F, **6**, in $CDCL_3$ 20
Figure S41. The HRESIMS spectrum of Tiuslactone F, **6**... 21

Figure S1. The ¹H-NMR spectrum of Tiuslactone A, **1**, in CDCL3

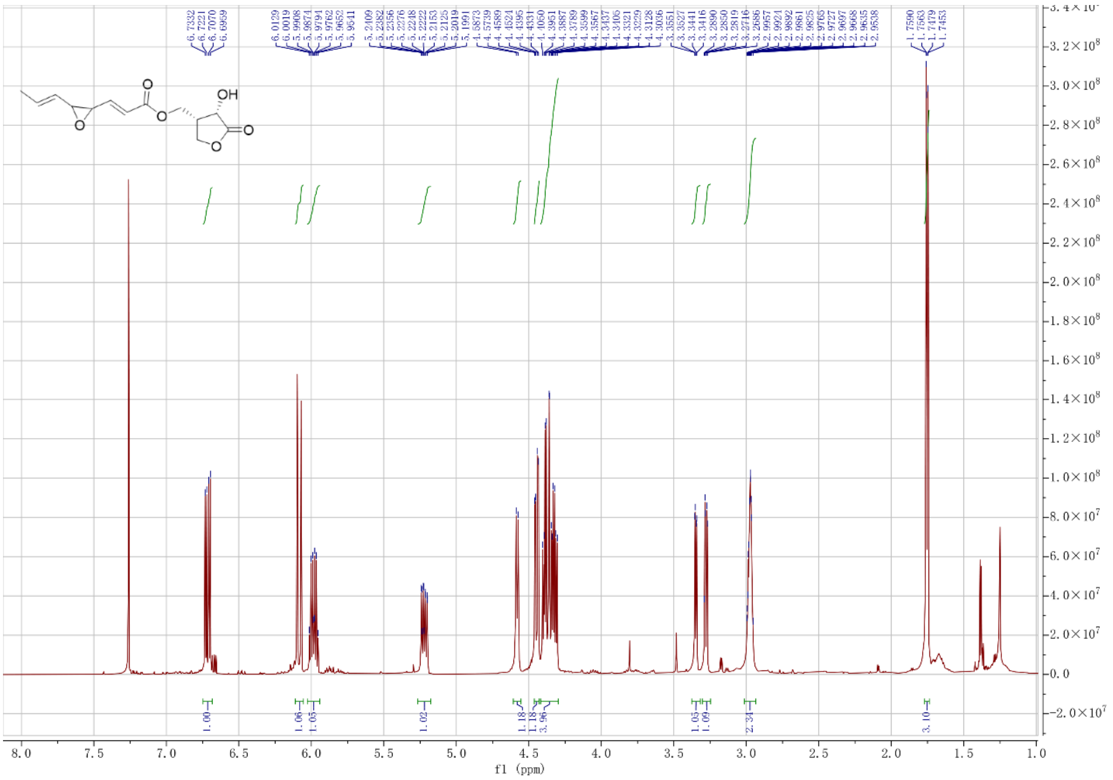

Figure S2. The ¹³C-NMR spectrum of Tiuslactone A, **1**, in CDCL3

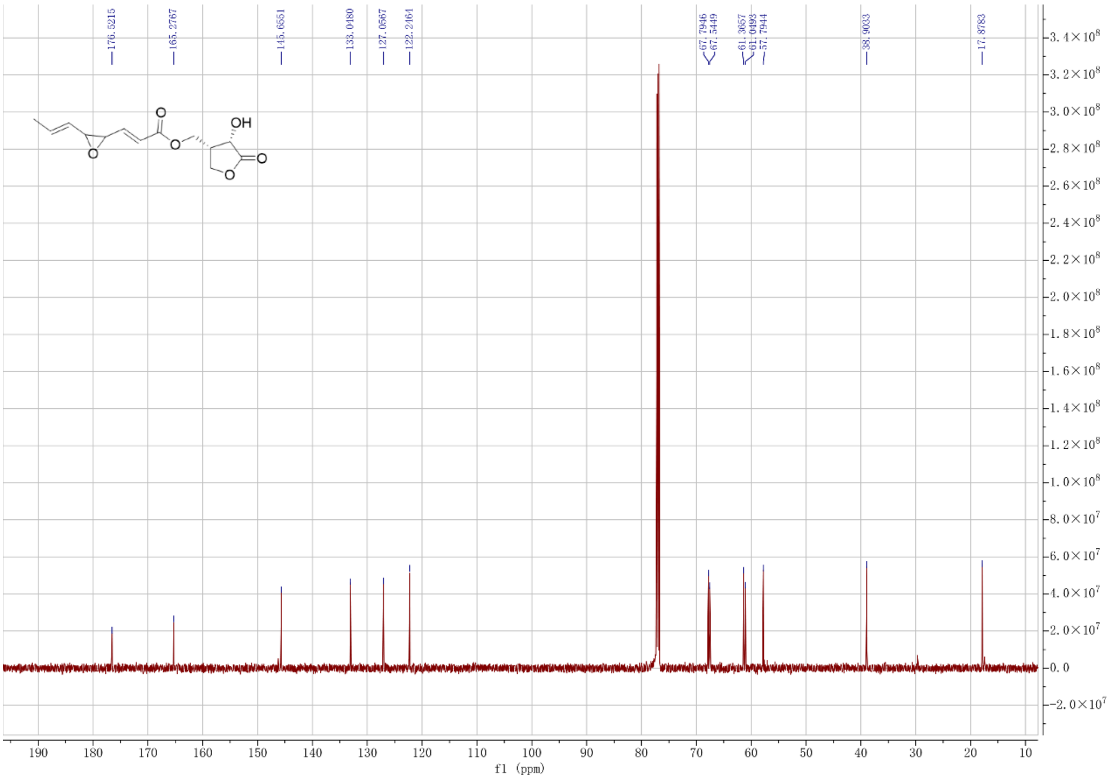

Figure S3. The HSQC spectrum of Tiuslactone A, **1**, in CDCL3

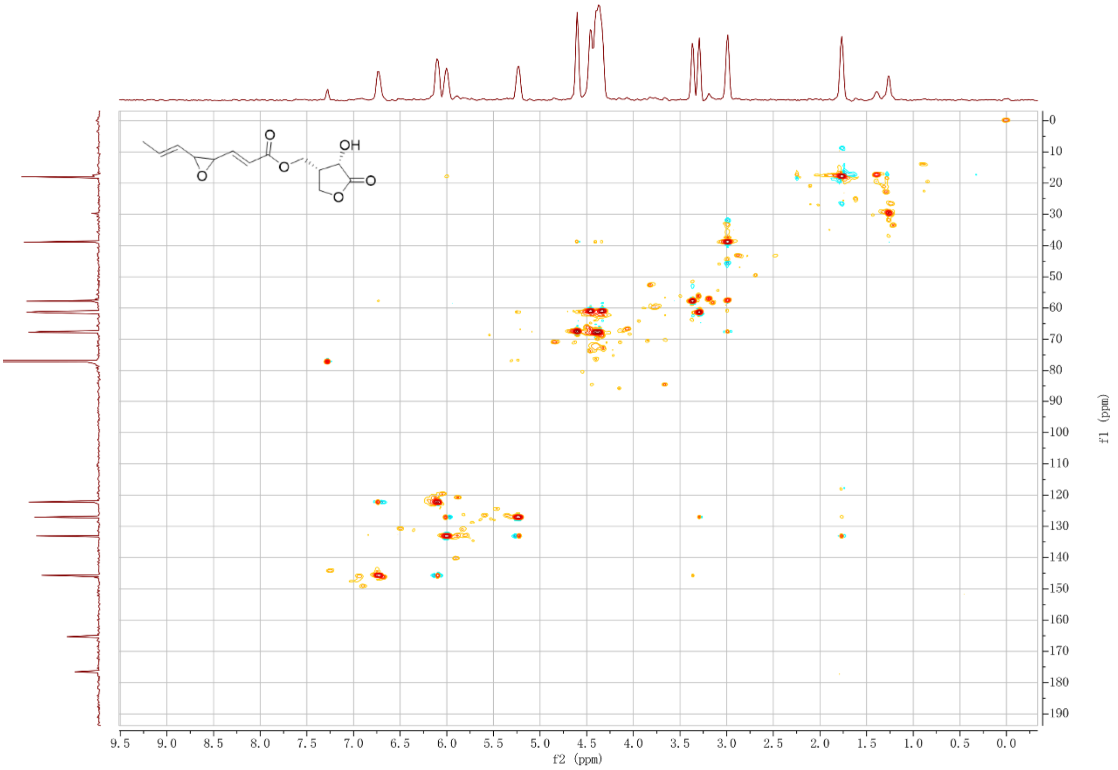

Figure S4. The HMBC spectrum of Tiuslactone A, **1**, in CDCL3

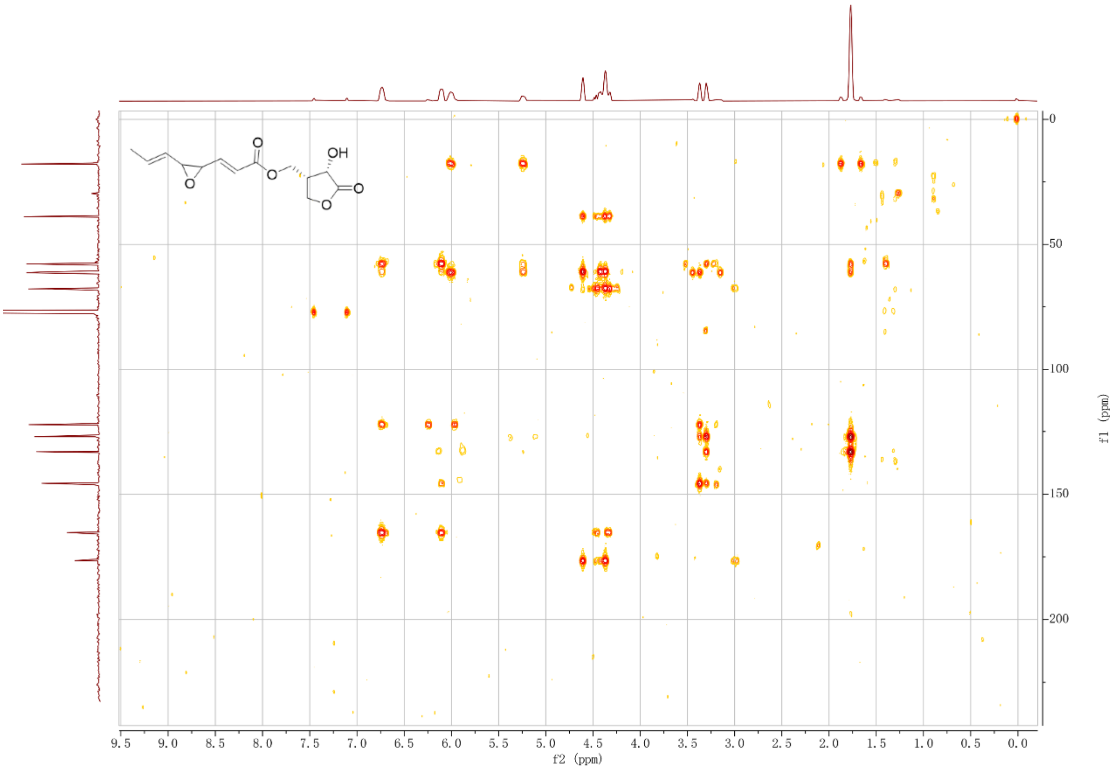

Figure S5. The ^{1}H-^{1}H COSY spectrum of Tiuslactone A, **1**, in CDCL$_3$

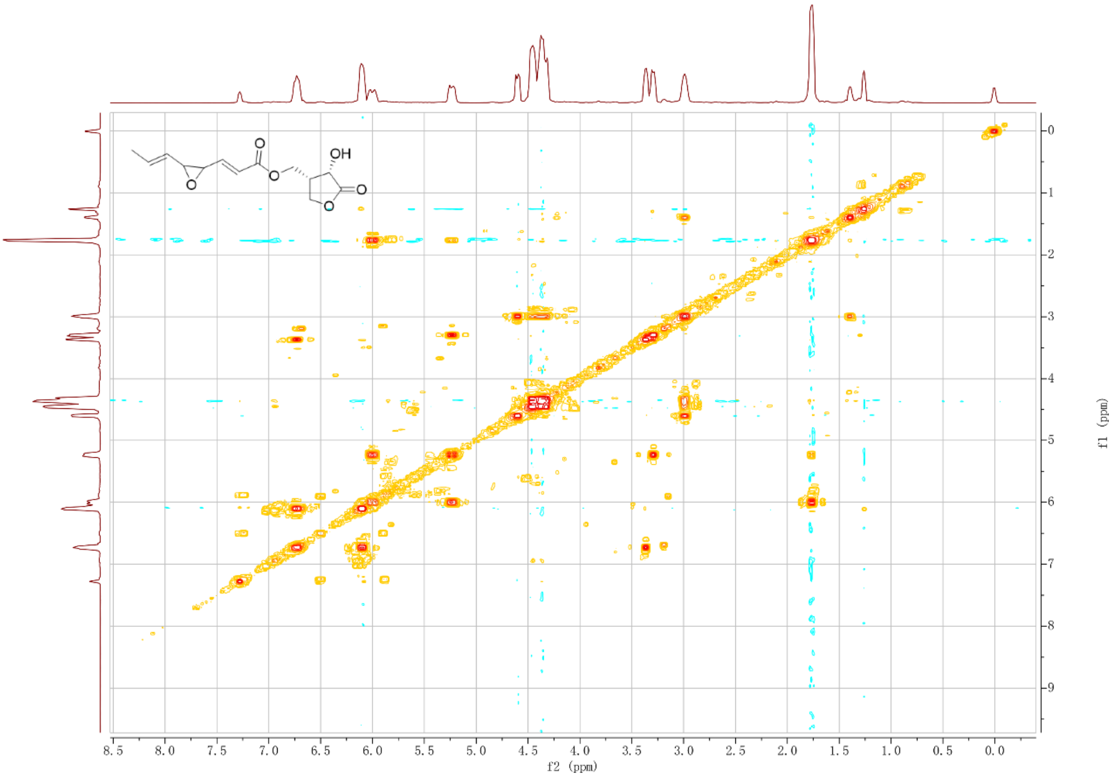

Figure S6. The NOESY spectrum of Tiuslactone A, **1**, in CDCL$_3$

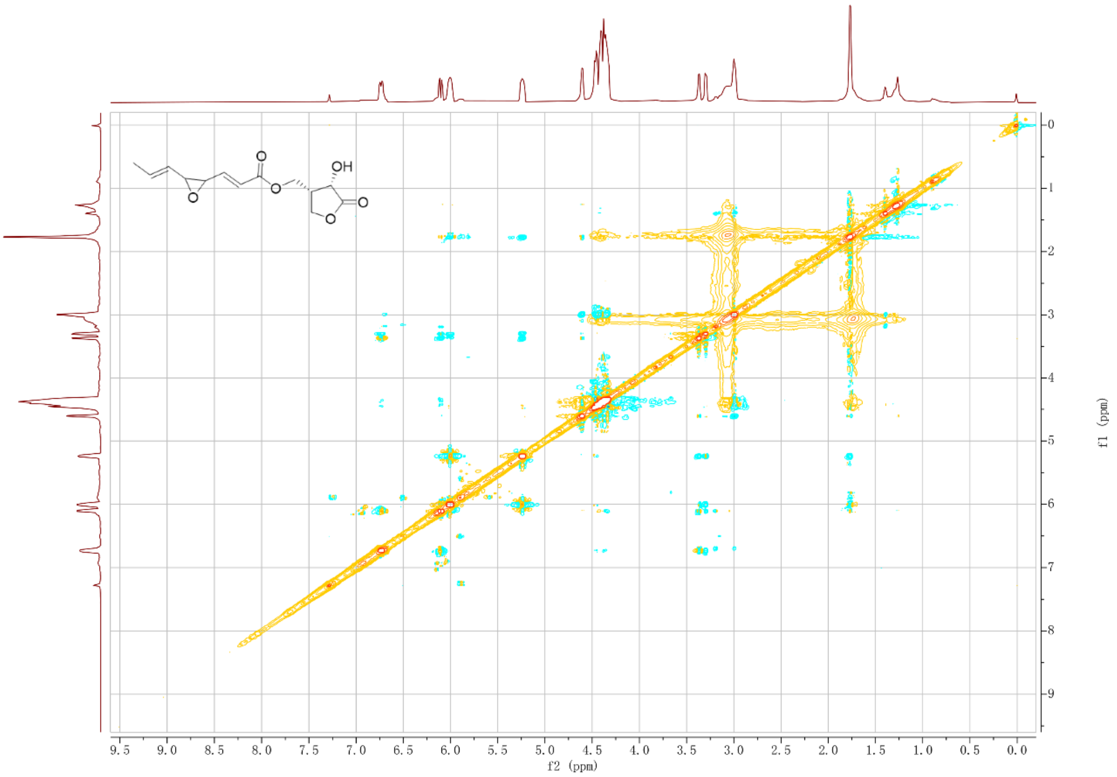

Figure S7. The HRESIMS spectrum of Tiuslactone A, **1**

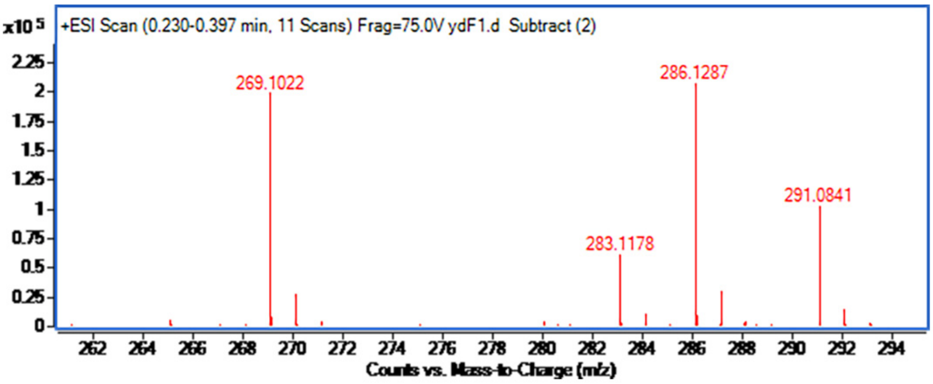

Figure S8. The ¹H-NMR spectrum of Tiuslactone B, **2**, in CDCL3

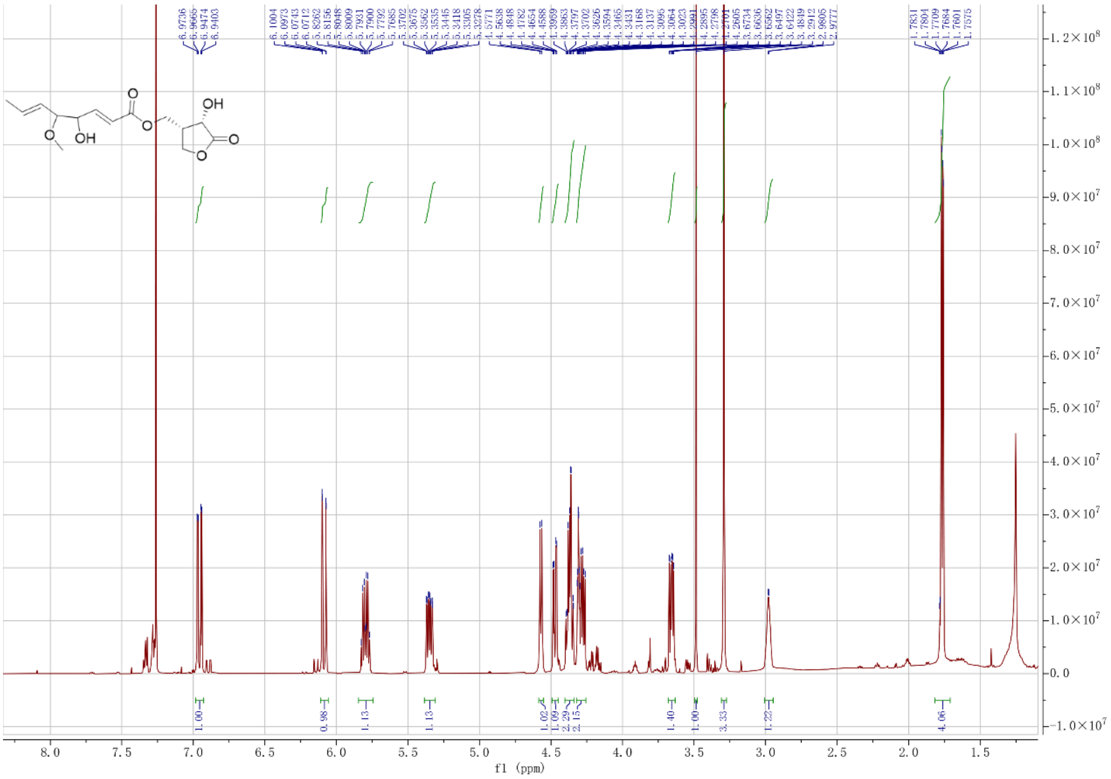

Figure S9. The ^{13}C-NMR spectrum of Tiuslactone B, **2**, in CDCL3

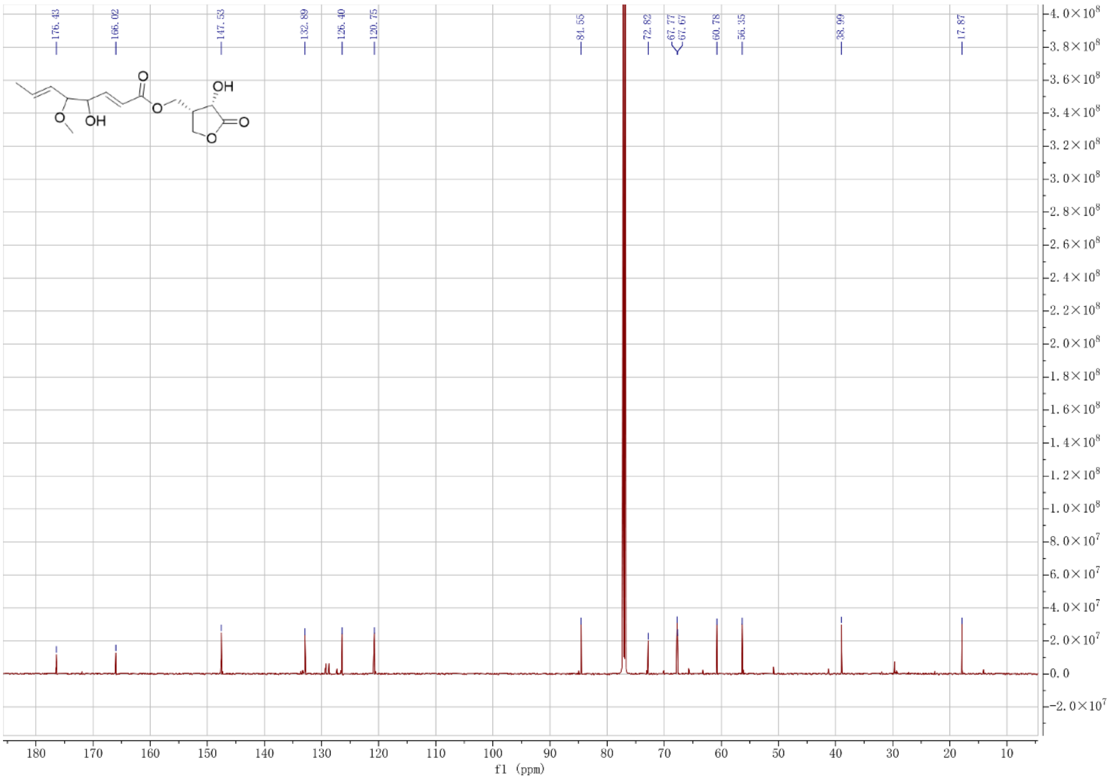

Figure S10. The HSQC spectrum of Tiuslactone B, **2**, in CDCL3

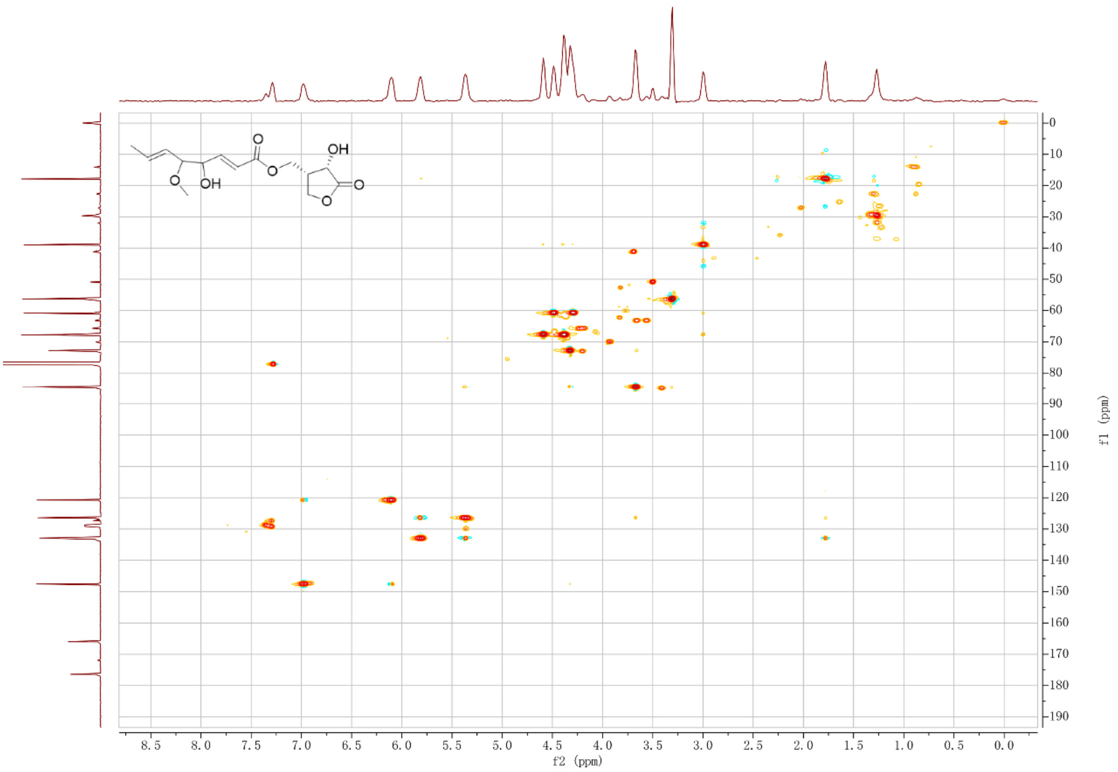

Figure S11. The HMBC spectrum of Tiuslactone B, **2**, in CDCL3

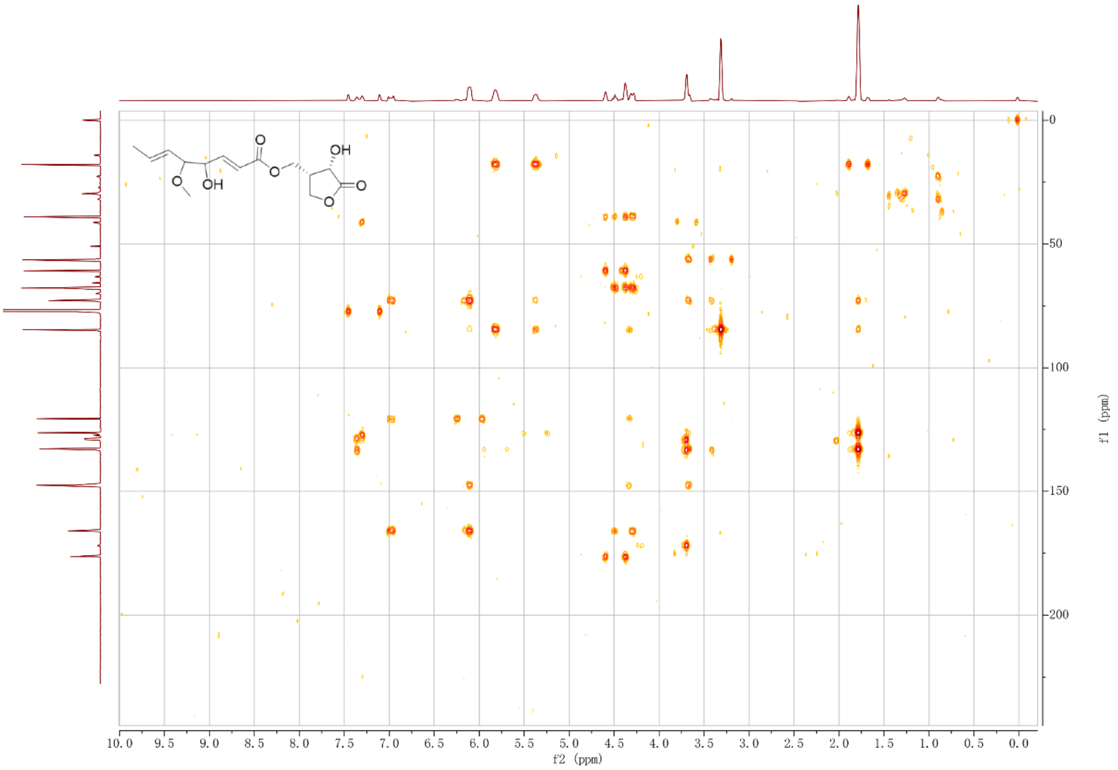

Figure S12. The ^{1}H-^{1}H COSY spectrum of Tiuslactone B, **2**, in CDCL3

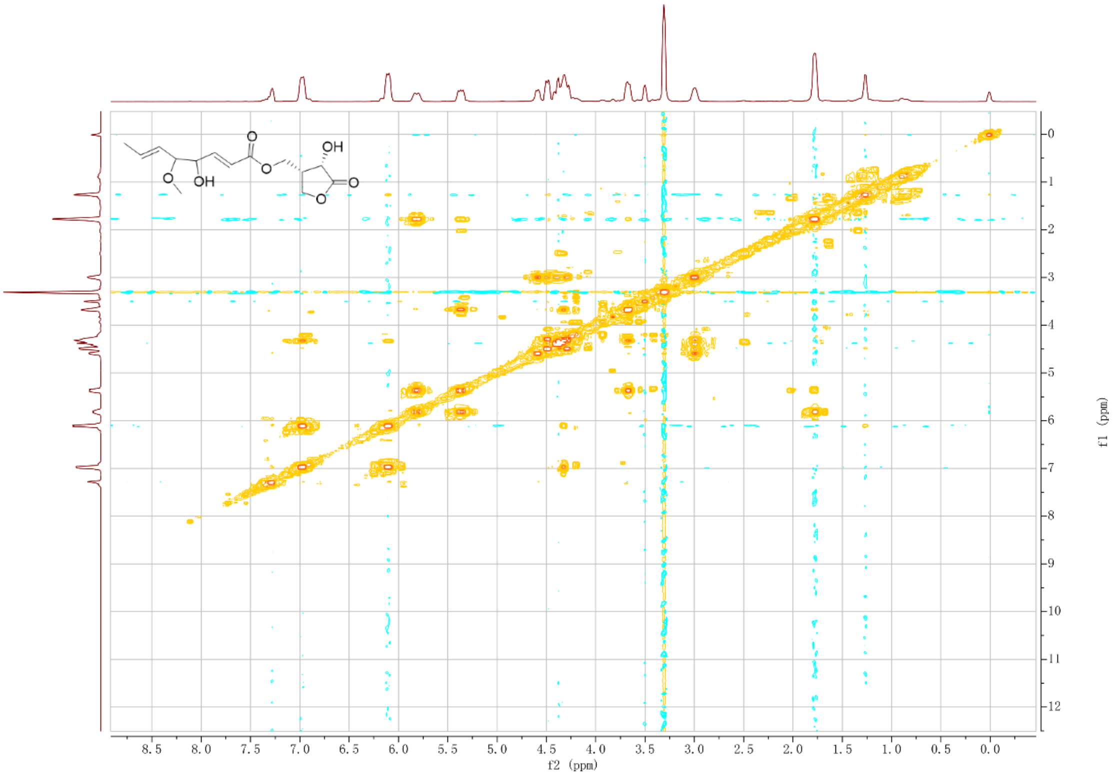

Figure S13. The NOESY spectrum of Tiuslactone B, **2**, in CDCL3

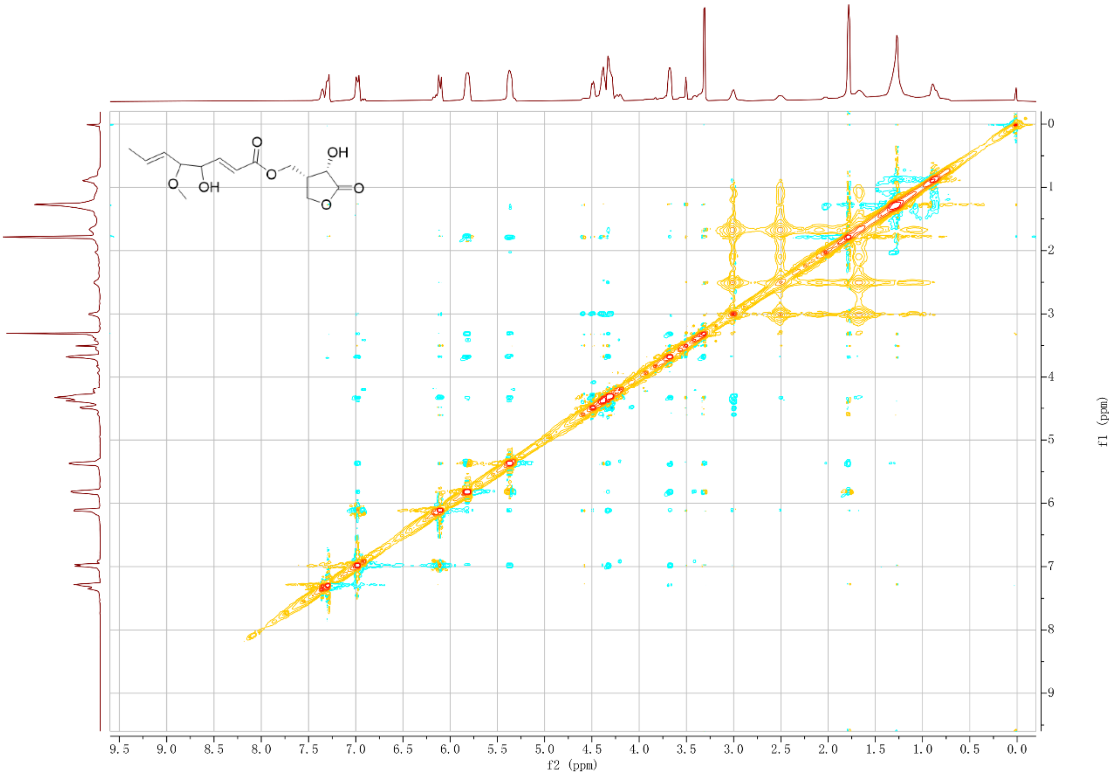

Figure S14. The HRESIMS spectrum of Tiuslactone B, **2**

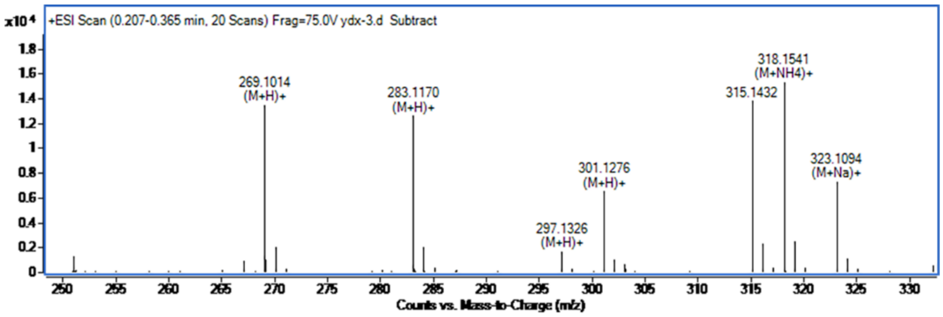

Figure S15. The ^{1}H-NMR spectrum of Tiuslactone C, **3**, in CDCL3

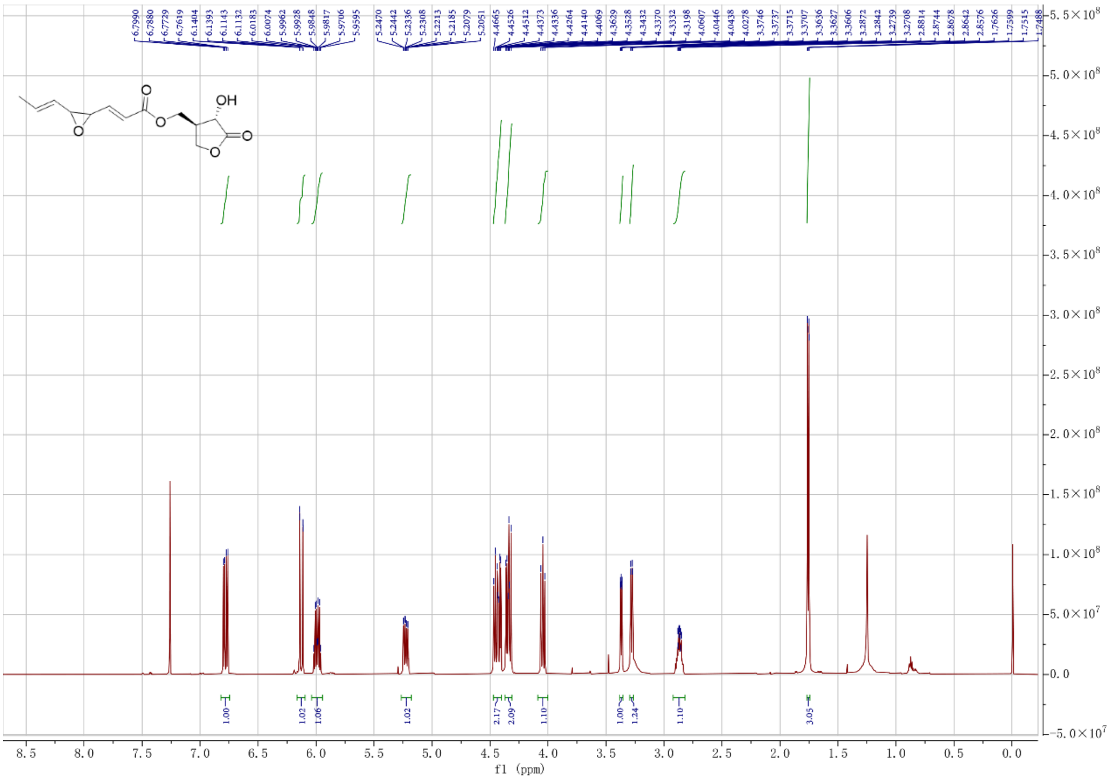

Figure S16. The ^{13}C-NMR spectrum of Tiuslactone C, **3**, in CDCL3

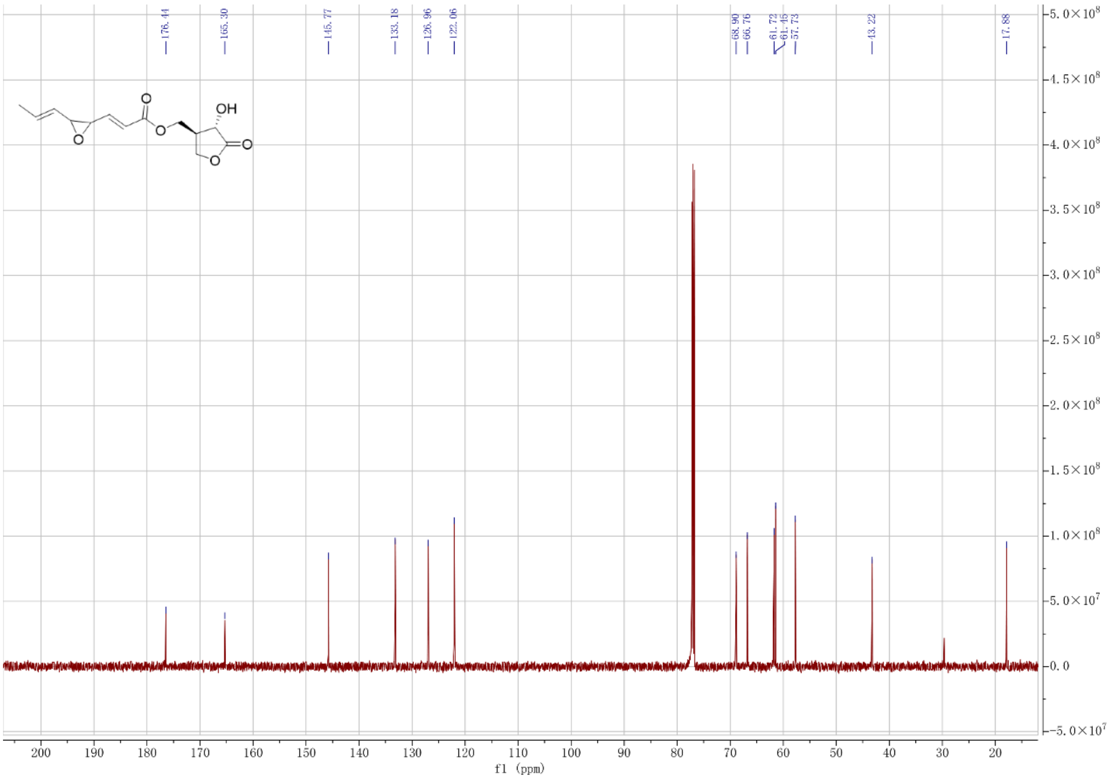

Figure S17. The HSQC spectrum of Tiuslactone C, **3**, in CDCL3

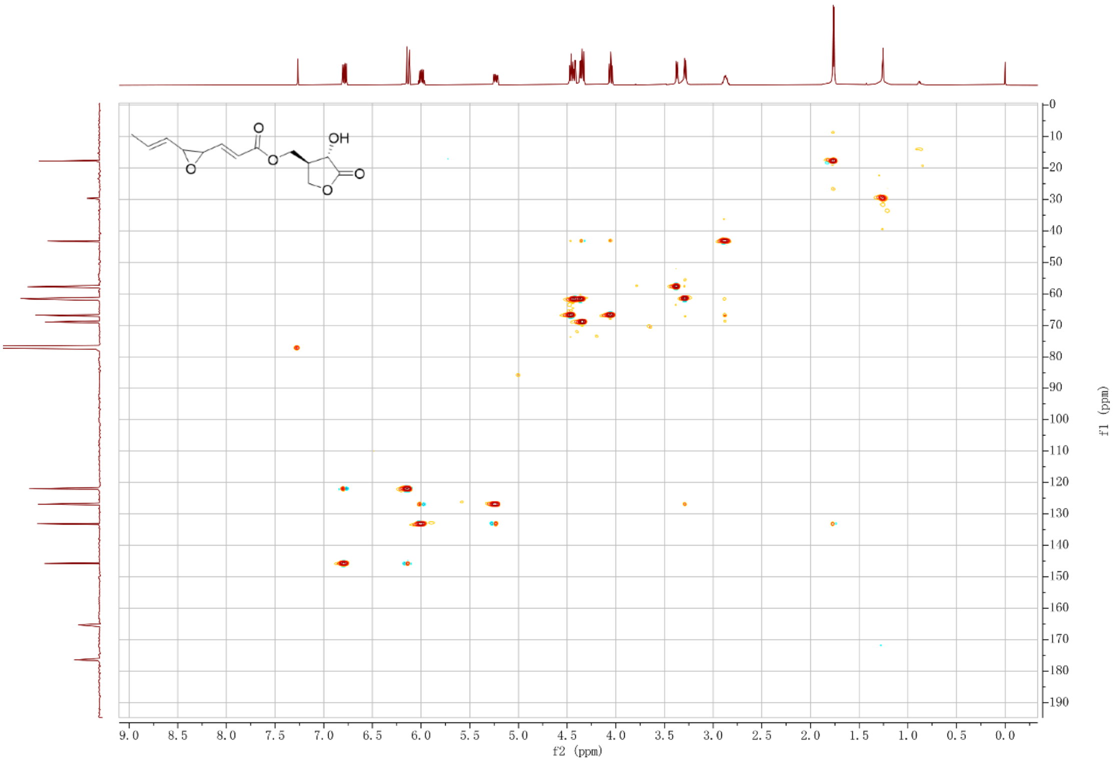

Figure S18. The HMBC spectrum of Tiuslactone C, **3**, in CDCL3

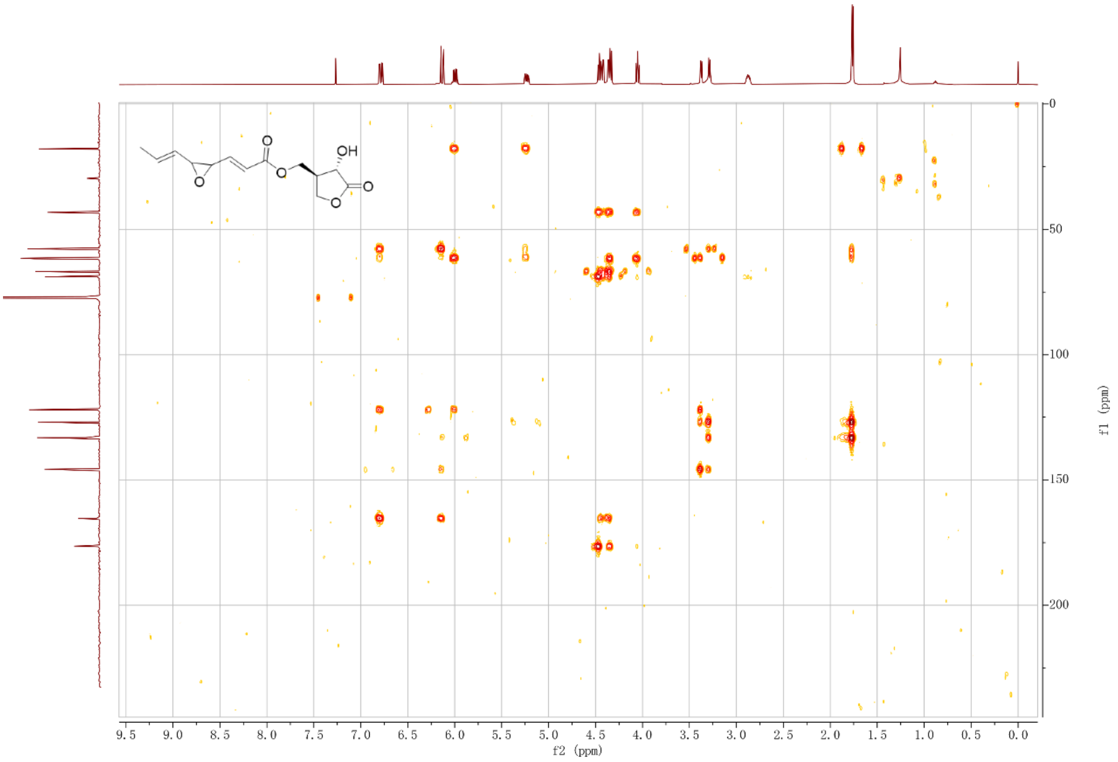

Figure S19. The ^{1}H-^{1}H COSY spectrum of Tiuslactone C, **3**, in CDCL$_3$

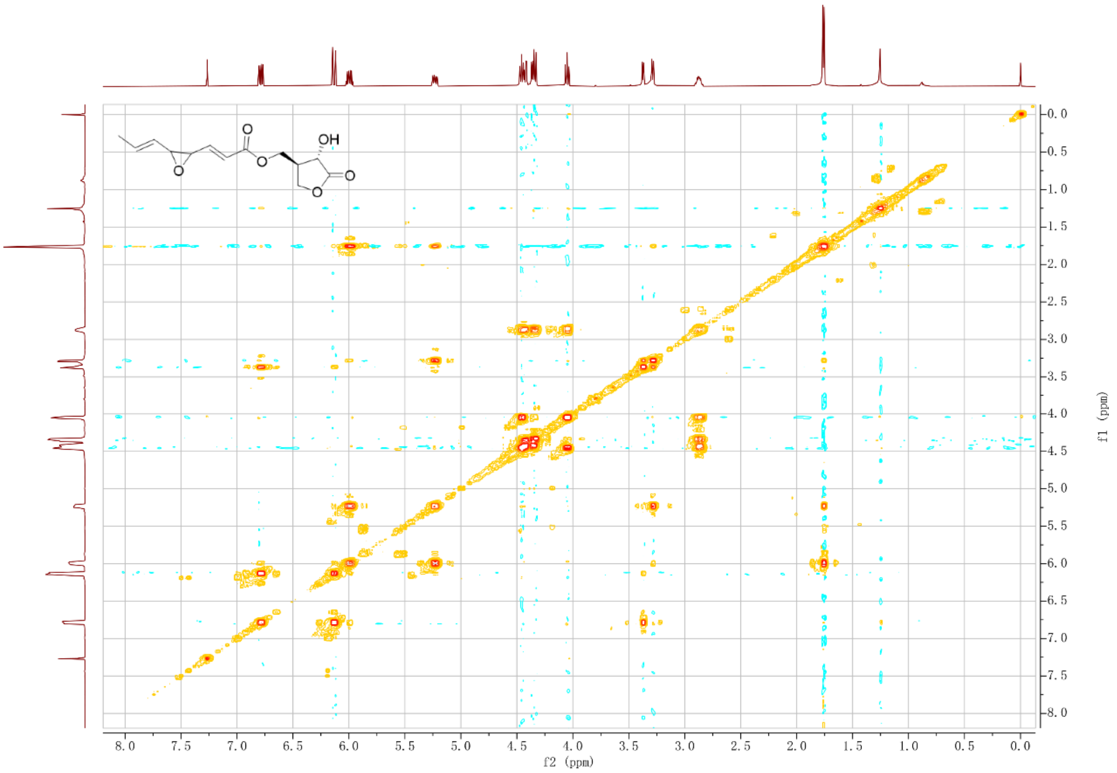

Figure S20. The HRESIMS spectrum of Tiuslactone C, **3**

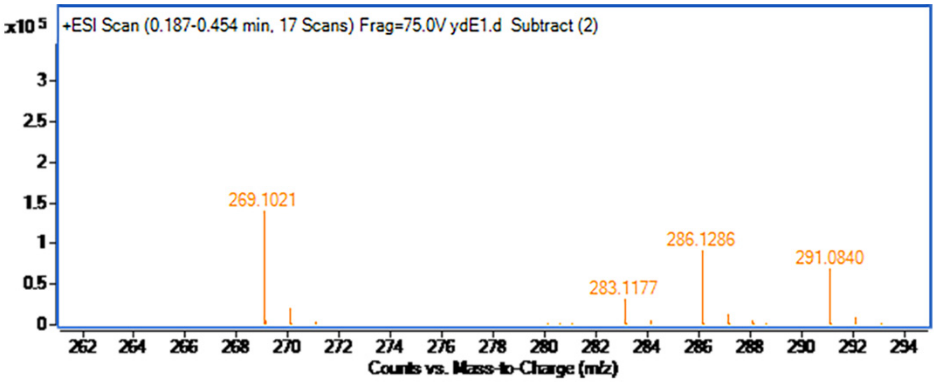

Figure S21. The ¹H-NMR spectrum of Tiuslactone D, **4**, in CDCL3

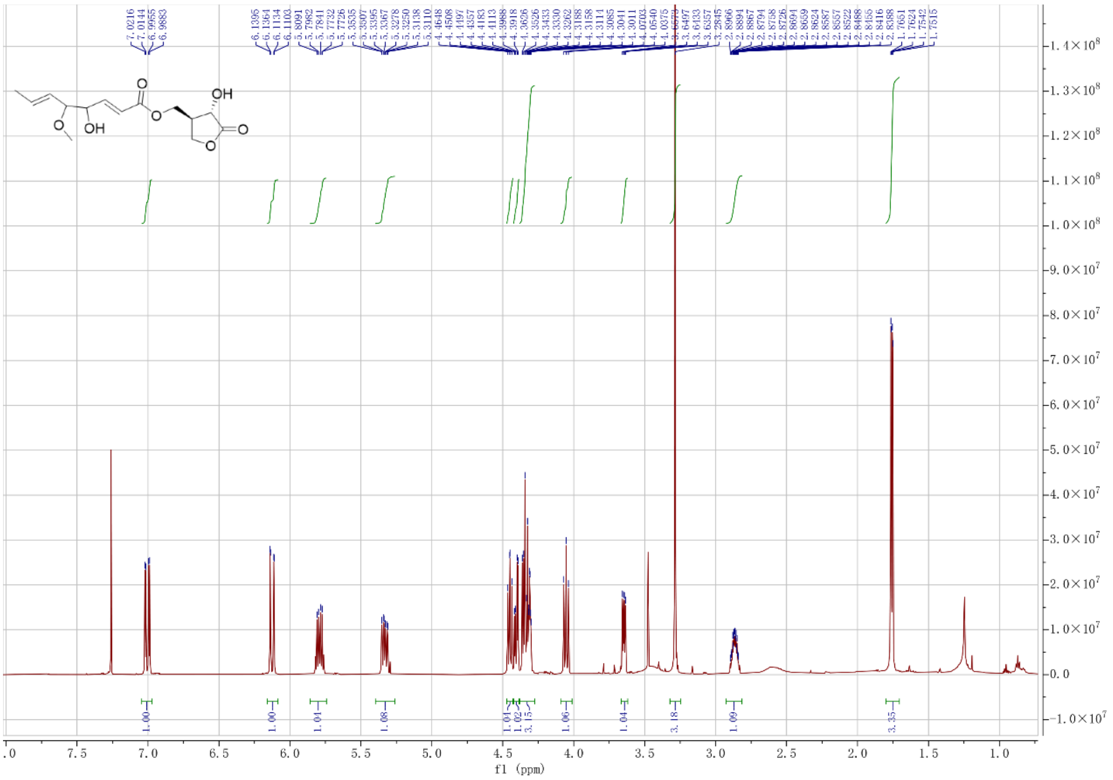

Figure S22. The ¹³C-NMR spectrum of Tiuslactone D, **4**, in CDCL3

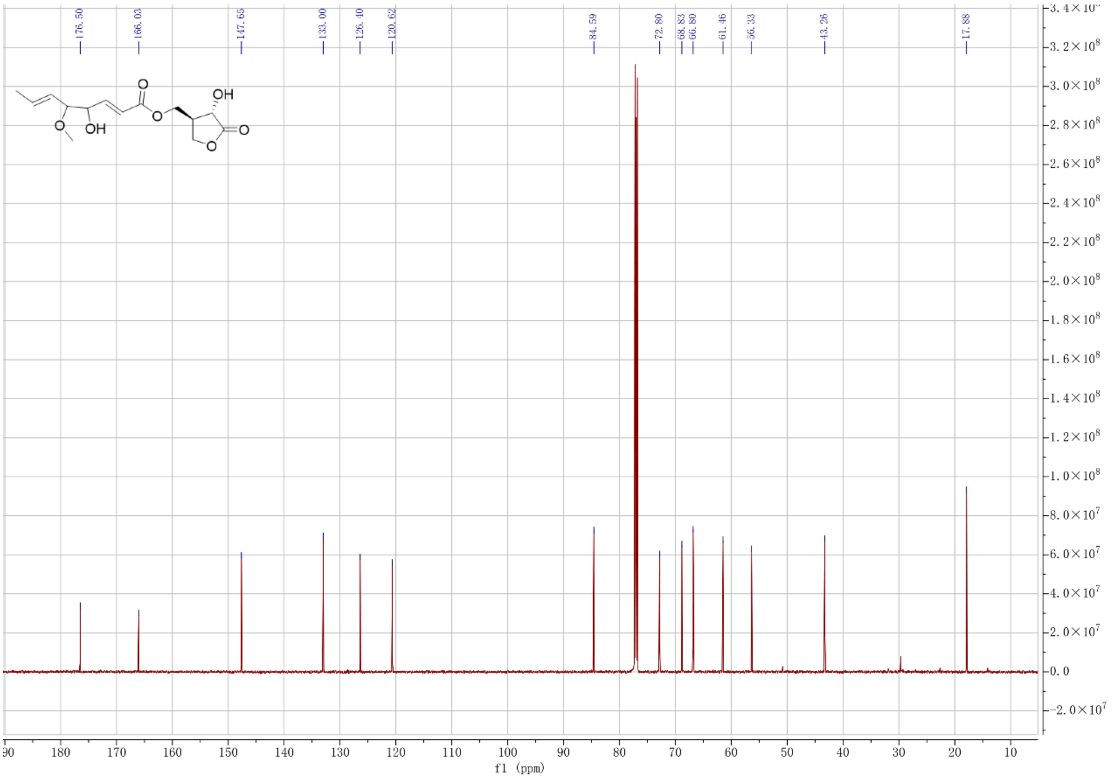

Figure S23. The HSQC spectrum of Tiuslactone D, **4**, in CDCL3

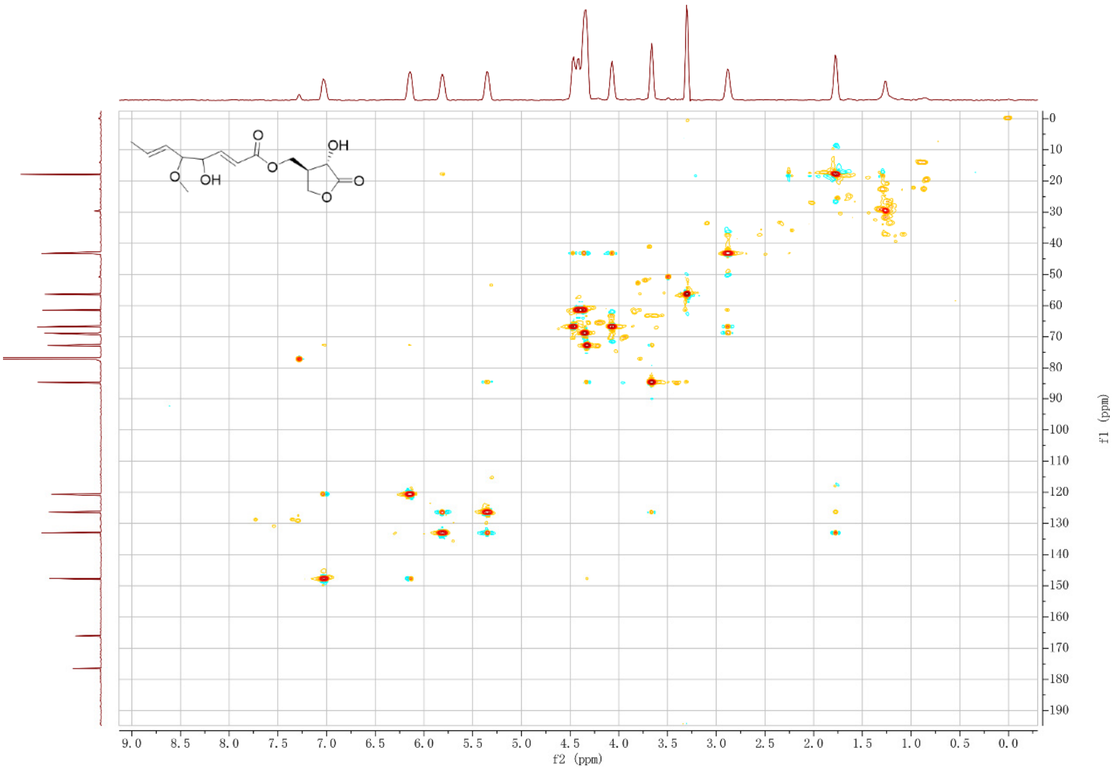

Figure S24. The HMBC spectrum of Tiuslactone D, **4**, in CDCL3

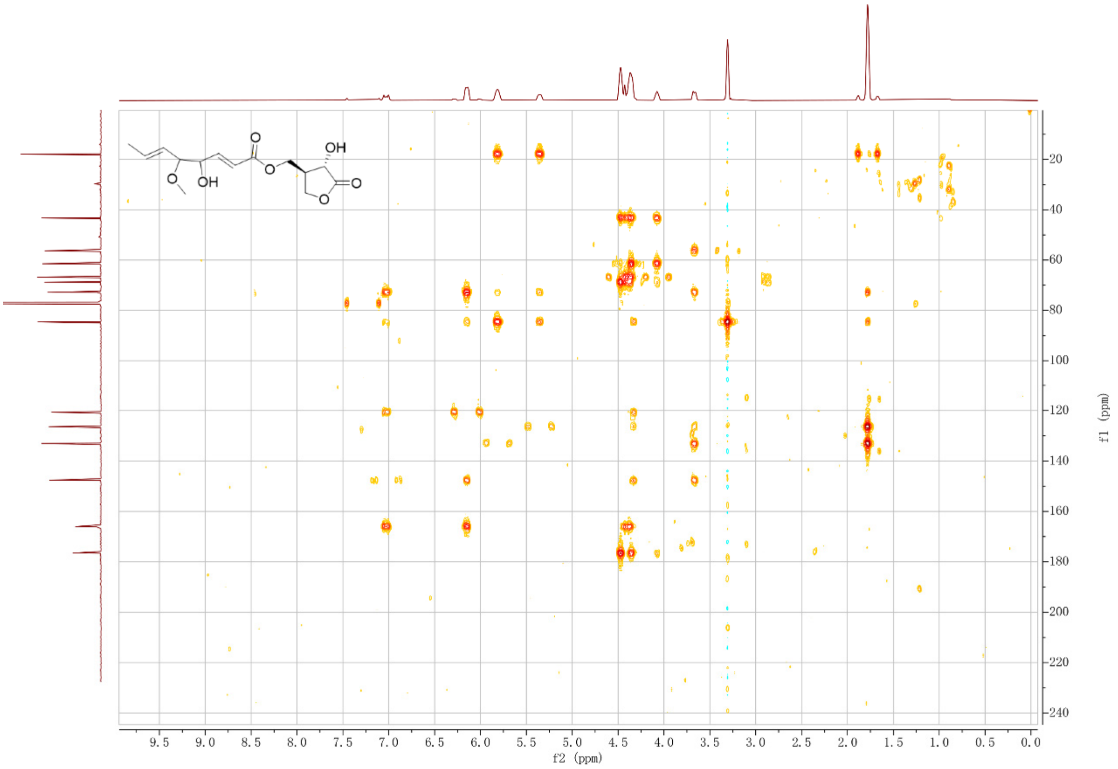

Figure S25. The ^{1}H-^{1}H COSY spectrum of Tiuslactone D, **4**, in CDCL3

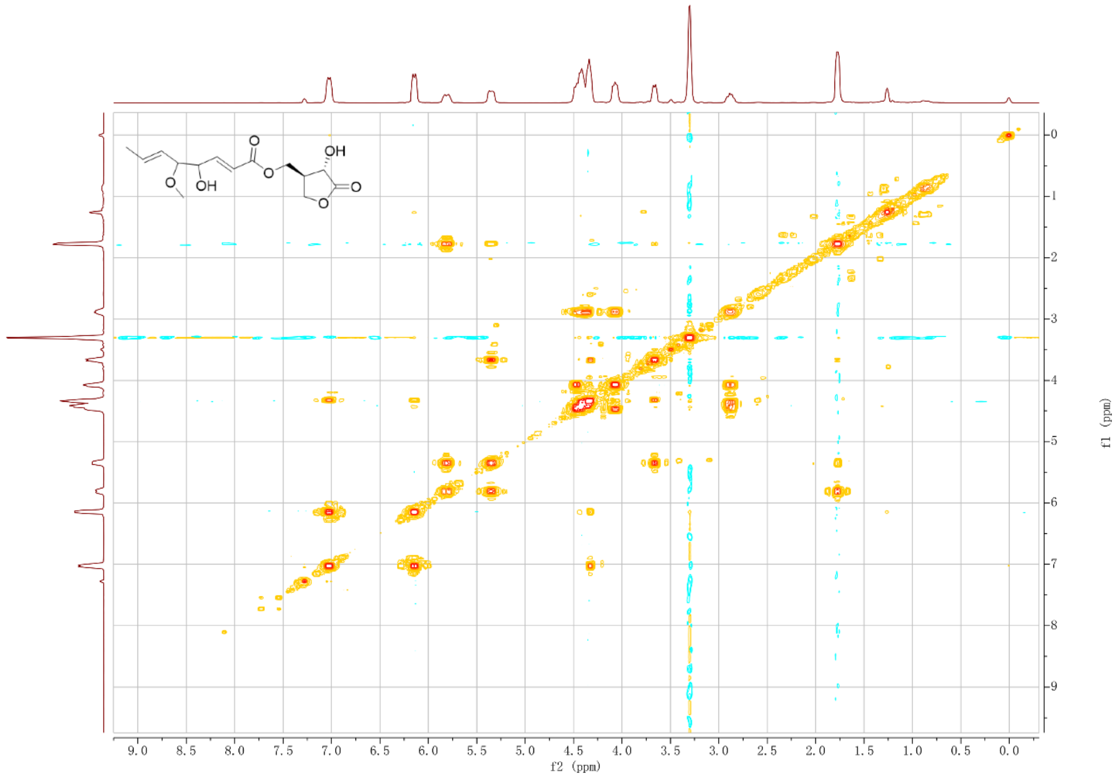

Figure S26. The NOESY spectrum of Tiuslactone D, **4**, in CDCL3

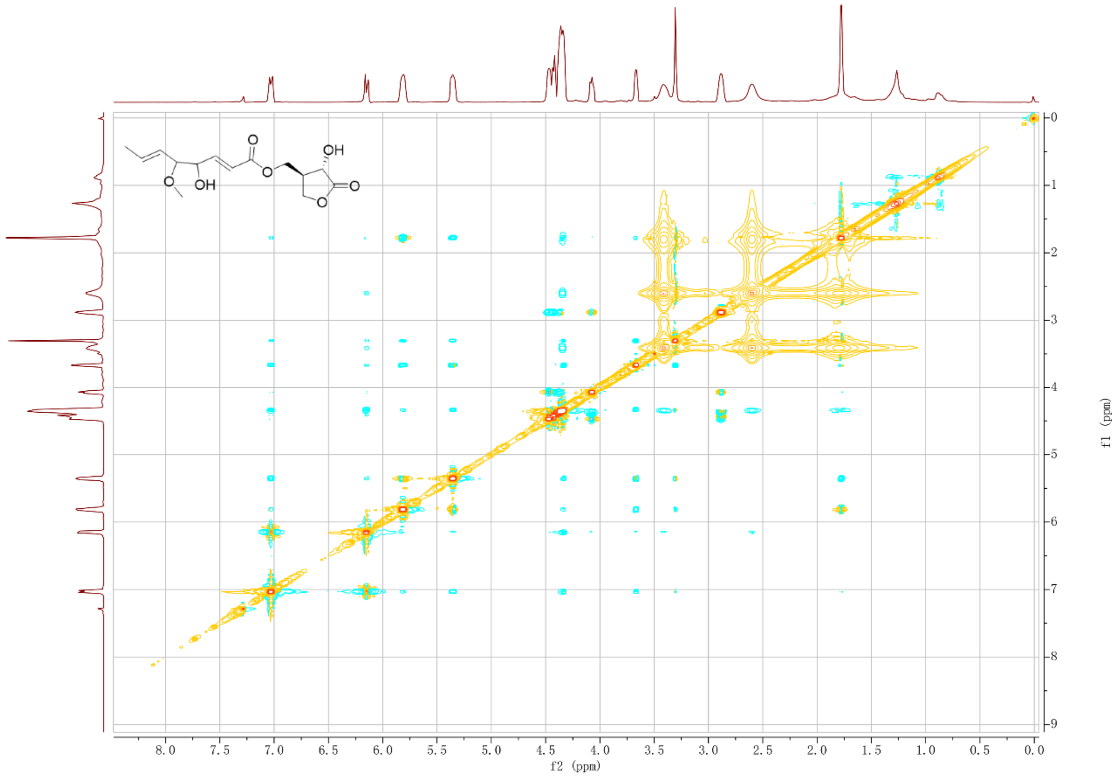

Figure S27. The HRESIMS spectrum of Tiuslactone D, **4**

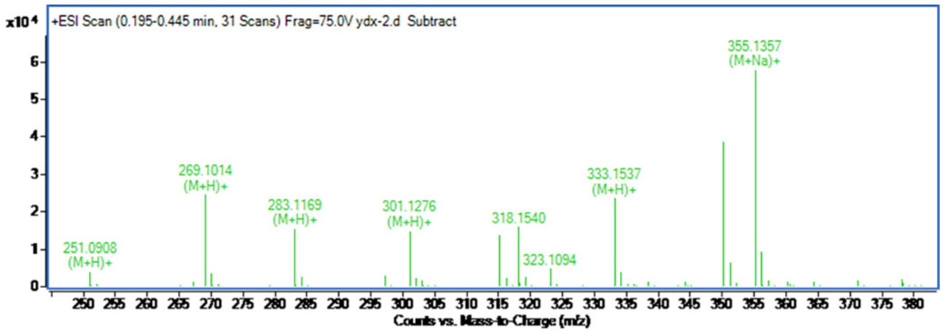

Figure S28. The ^{1}H-NMR spectrum of Tiuslactone E, **5**, in CDCL3

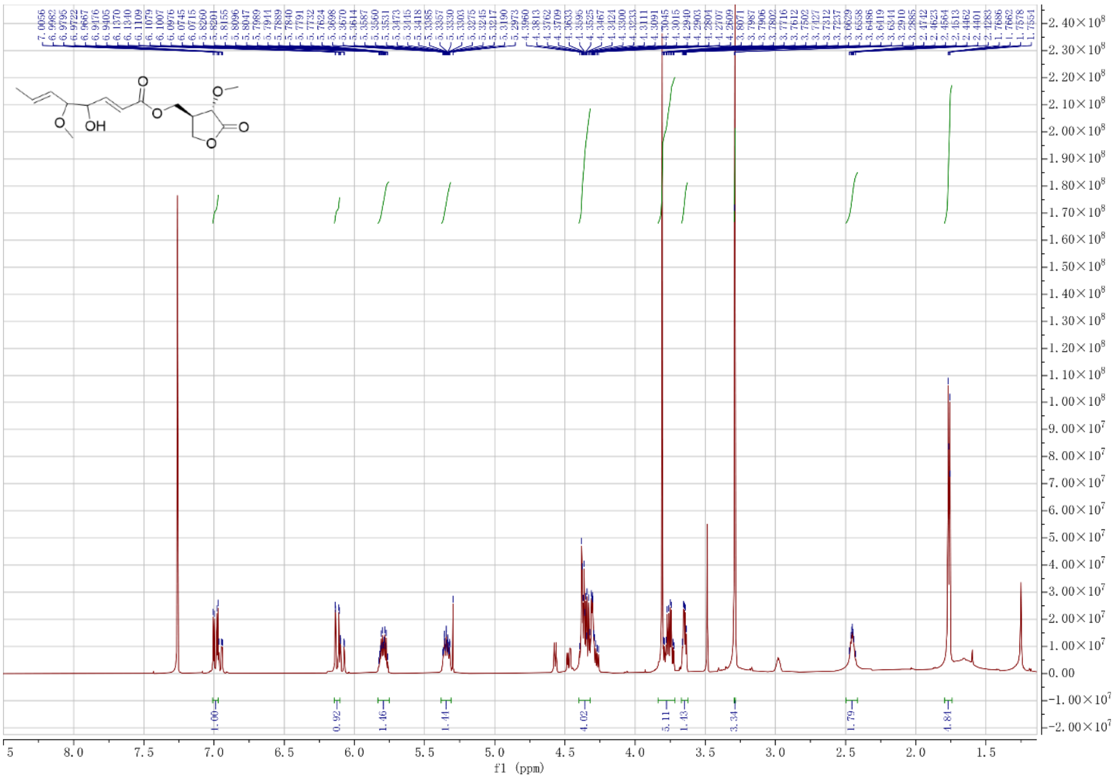

Figure S29. The 13C-NMR spectrum of Tiuslactone E, **5**, in CDCL3

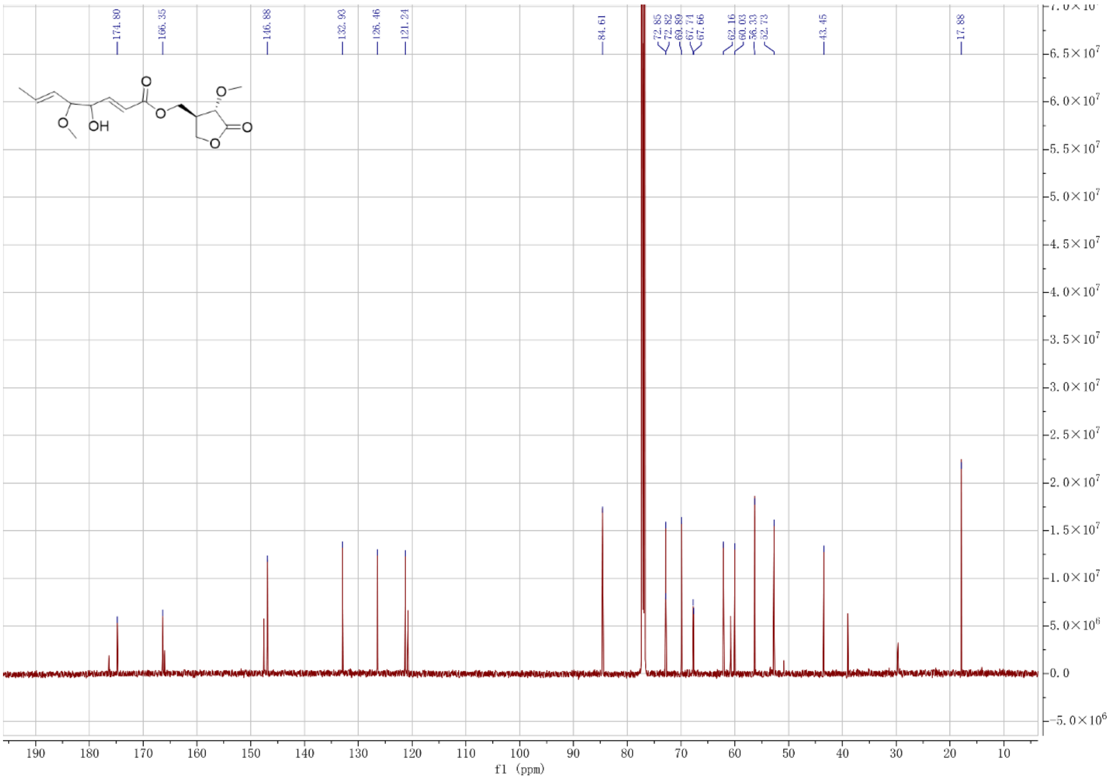

Figure S30. The HSQC spectrum of Tiuslactone E, **5**, in CDCL3

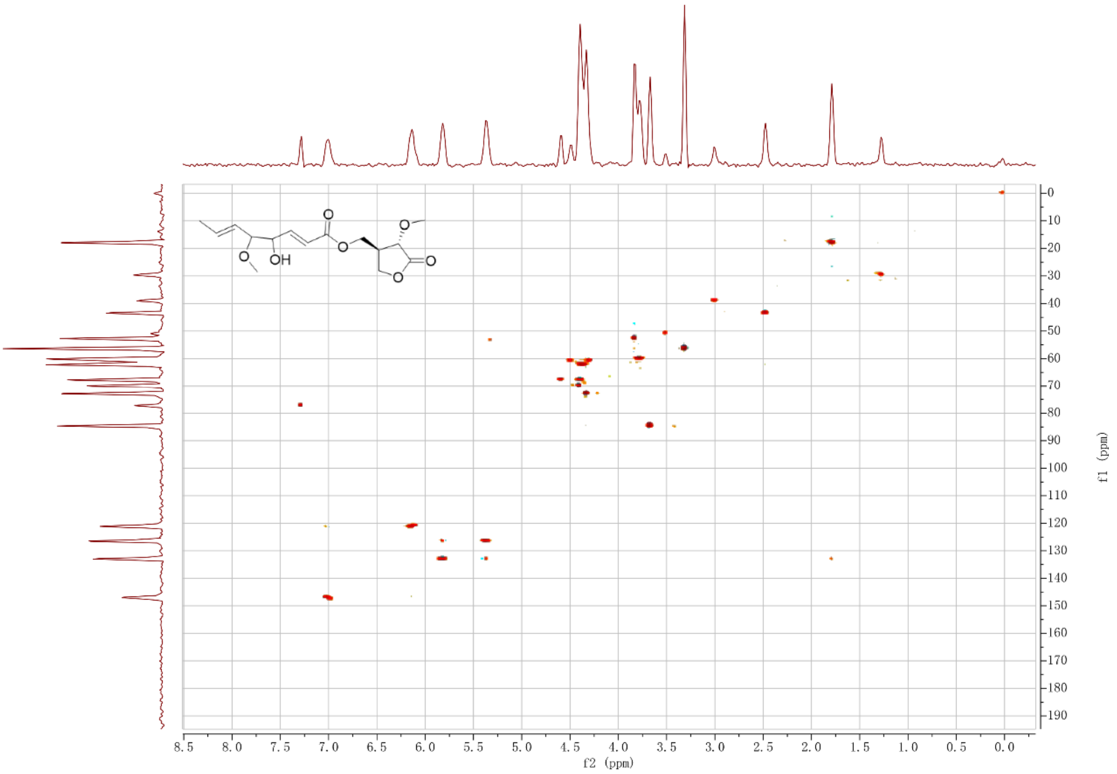

Figure S31. The HMBC spectrum of Tiuslactone E, **5**, in CDCL3

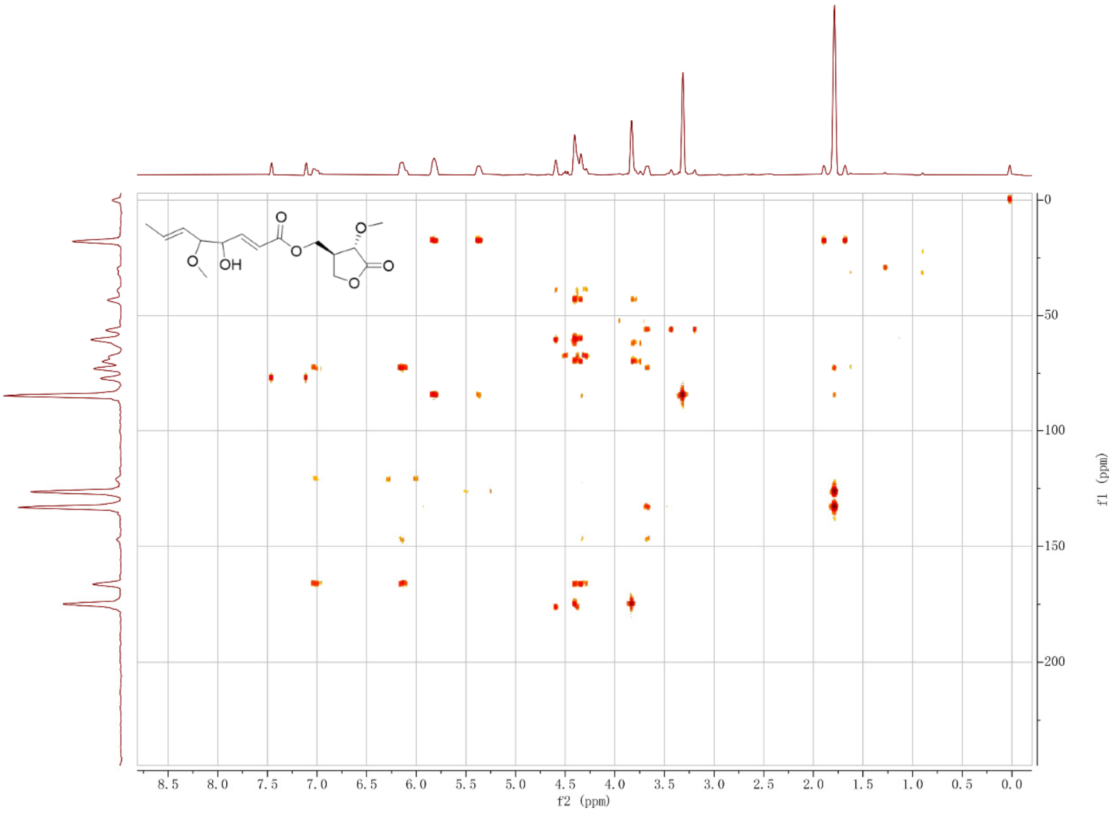

Figure S32. The ^{1}H-^{1}H COSY spectrum of Tiuslactone E, **5**, in CDCL3

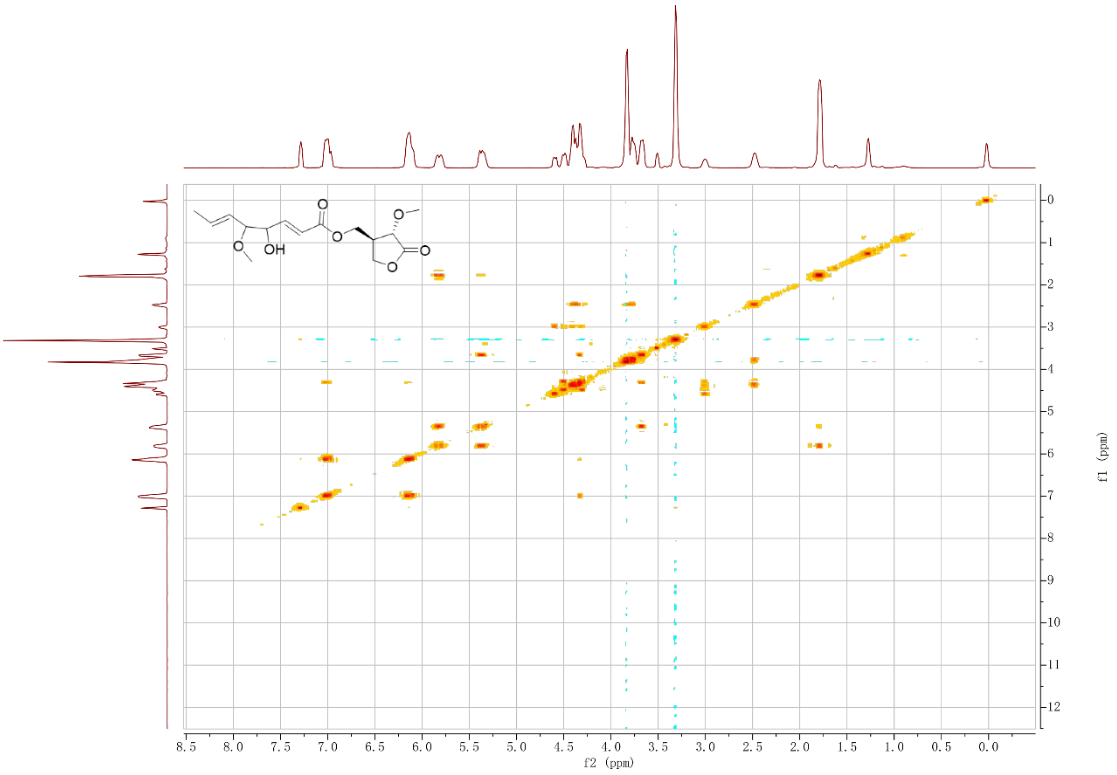

Figure S33. The NOESY spectrum of Tiuslactone E, **5**, in CDCL3

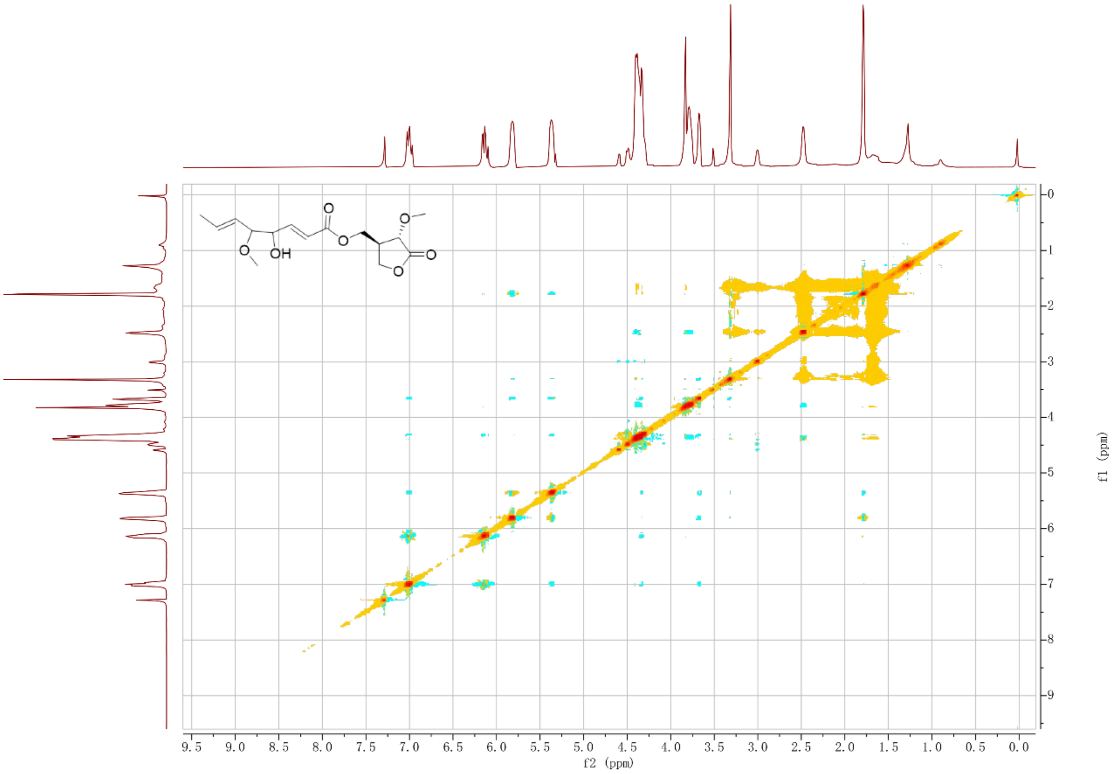

Figure S34. The HRESIMS spectrum of Tiuslactone E, **5**

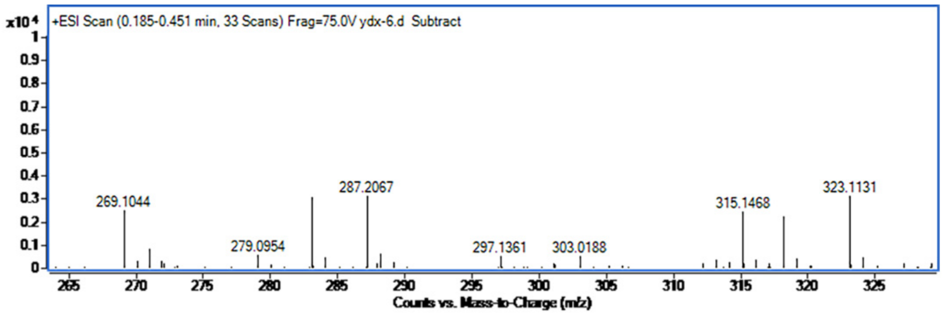

Figure S35. The ^{1}H-NMR spectrum of Tiuslactone F, **6**, in CDCL3

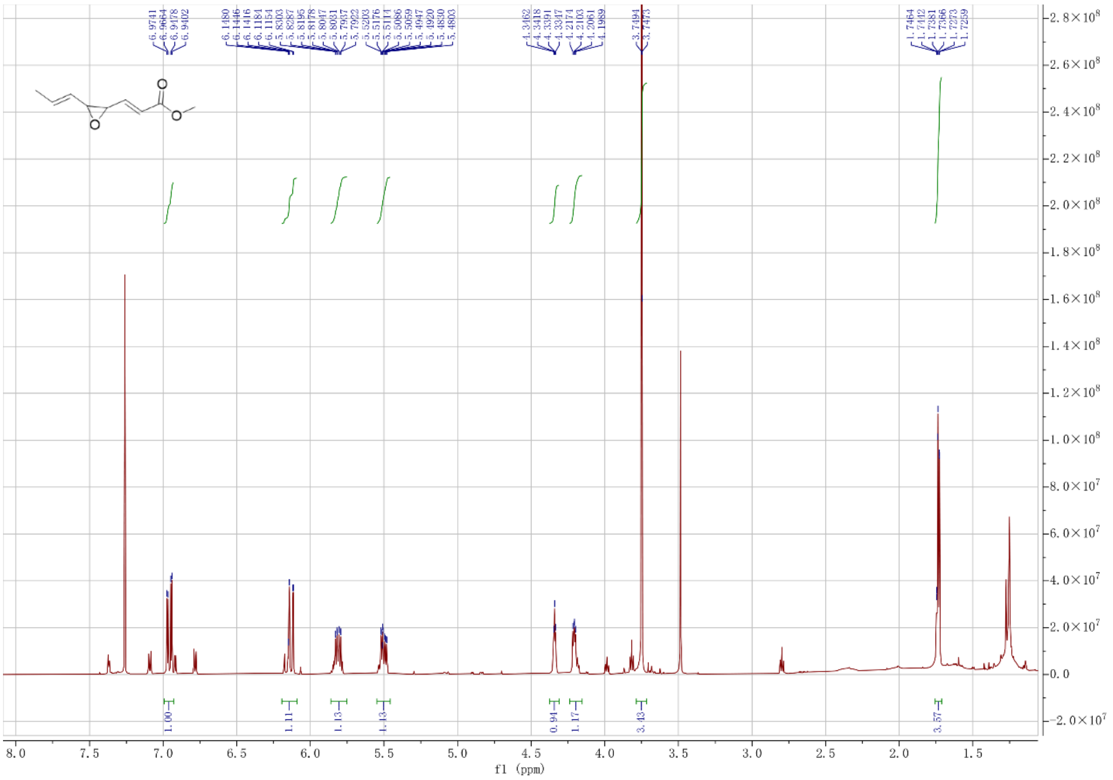

Figure S36. The ^{13}C-NMR spectrum of Tiuslactone F, **6**, in CDCL3

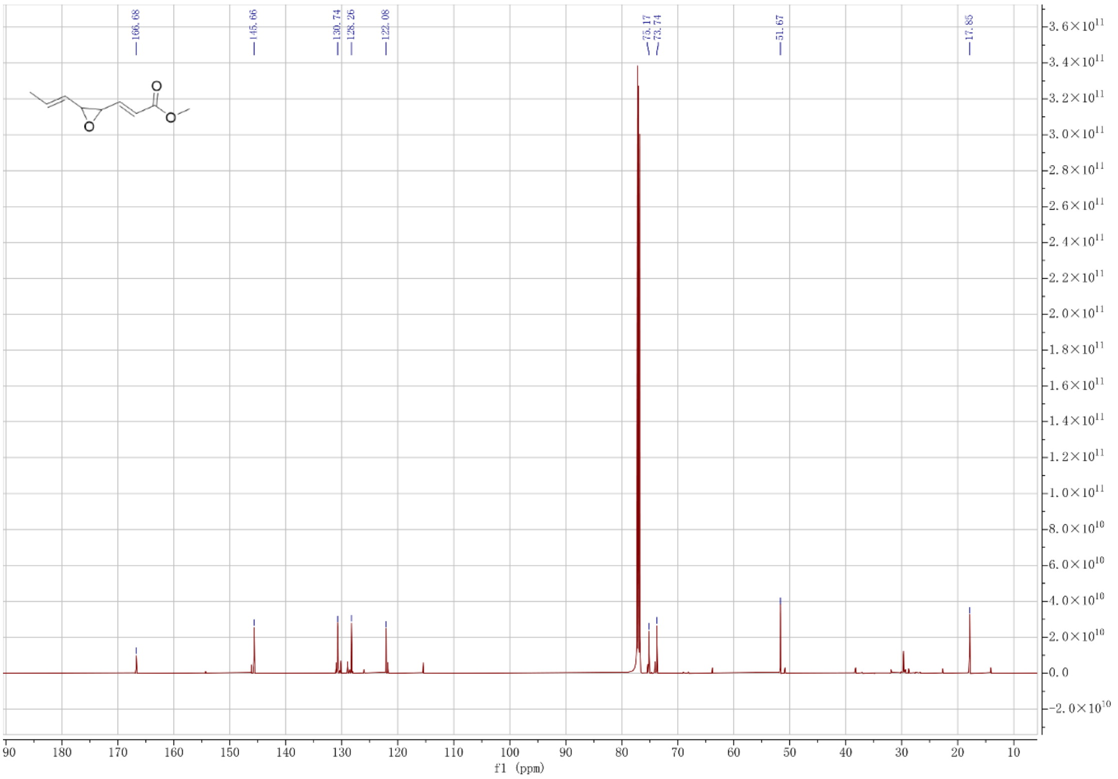

Figure S37. The HSQC spectrum of Tiuslactone F, **6**, in CDCL3

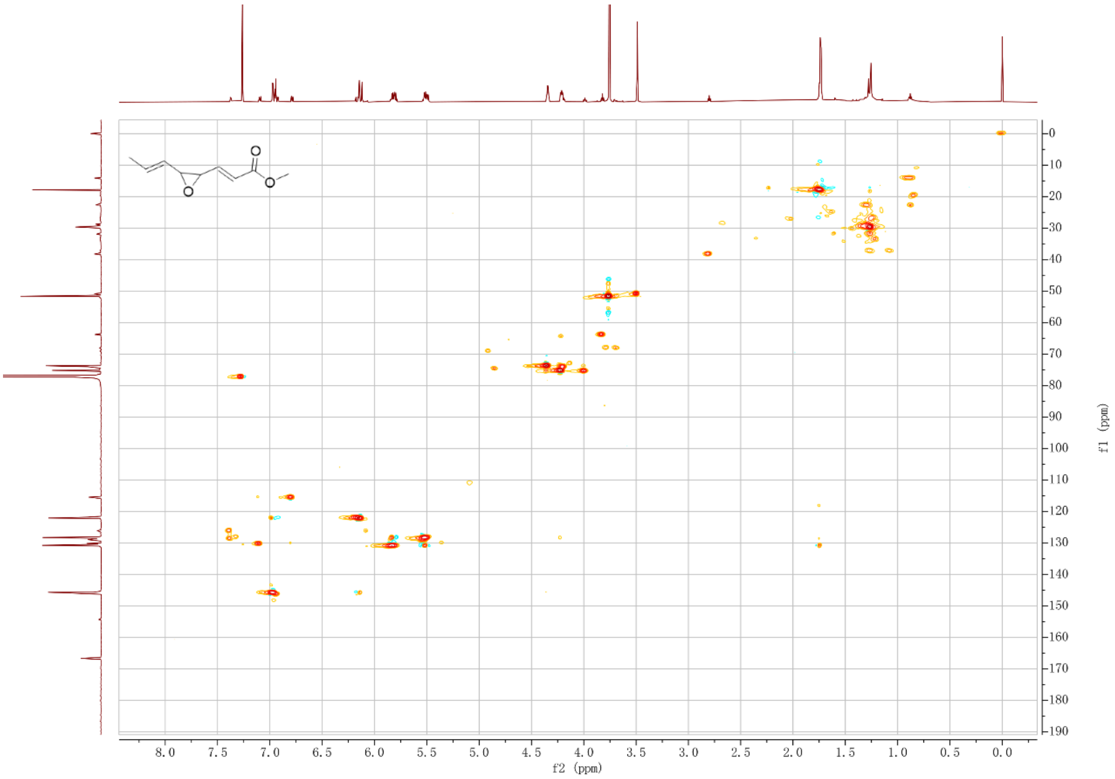

Figure S38. The HMBC spectrum of Tiuslactone F, **6**, in CDCL3

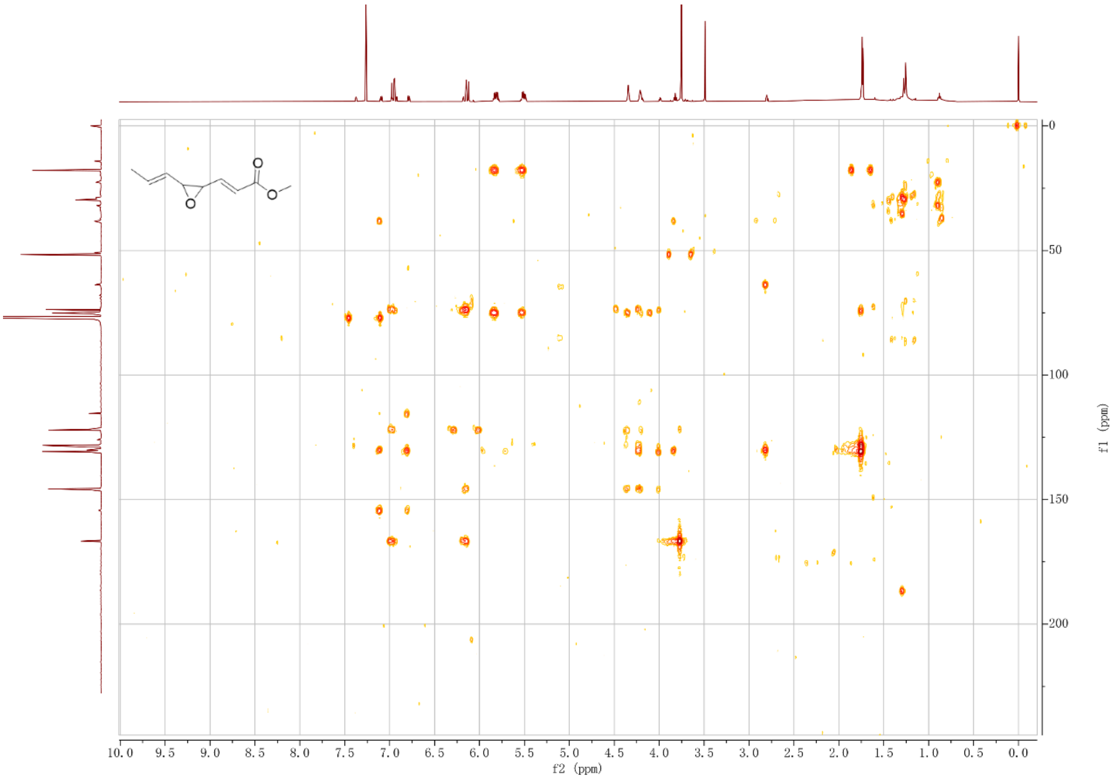

Figure S39. The ^{1}H-^{1}H COSY spectrum of Tiuslactone F, **6**, in CDCL3

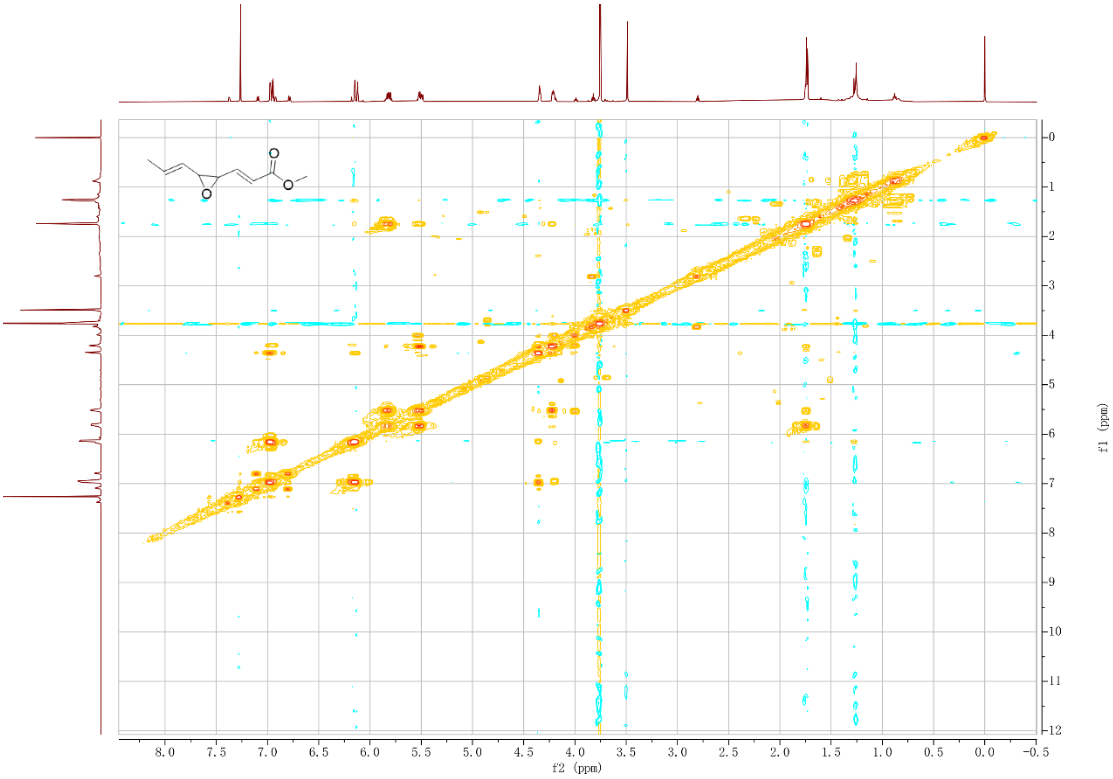

Figure S40. The NOESY spectrum of Tiuslactone F, **6**, in CDCL3

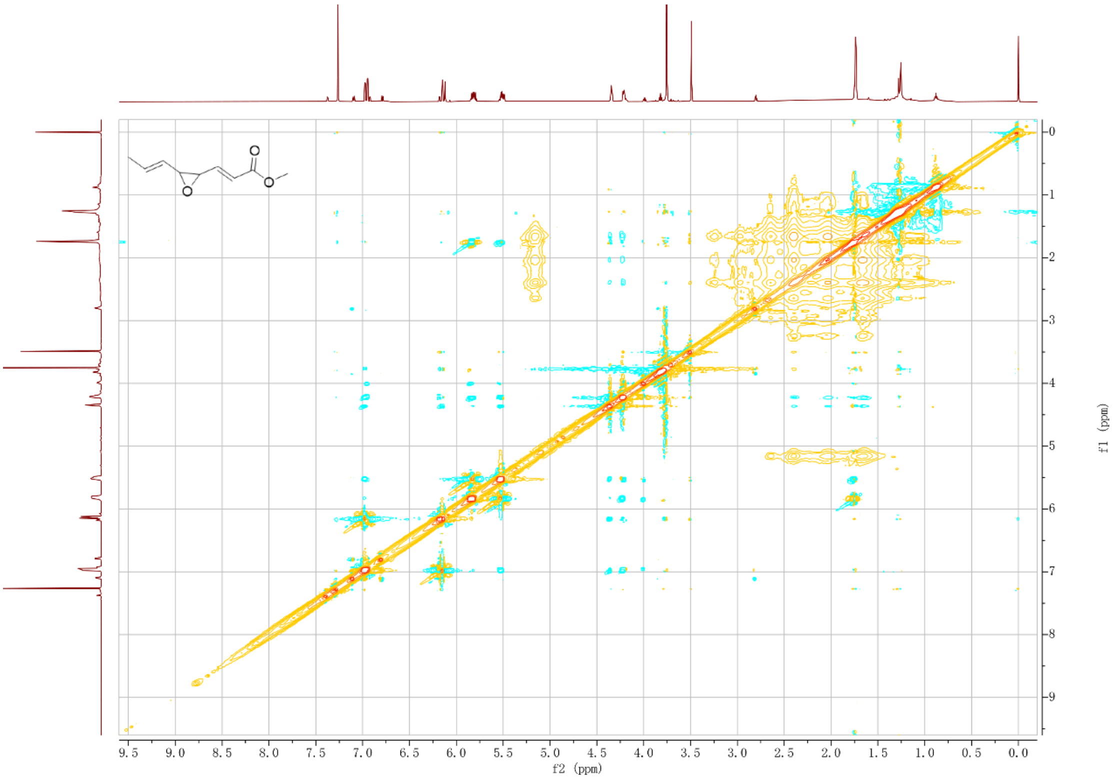

Figure S41. The HRESIMS spectrum of Tiuslactone F, **6**

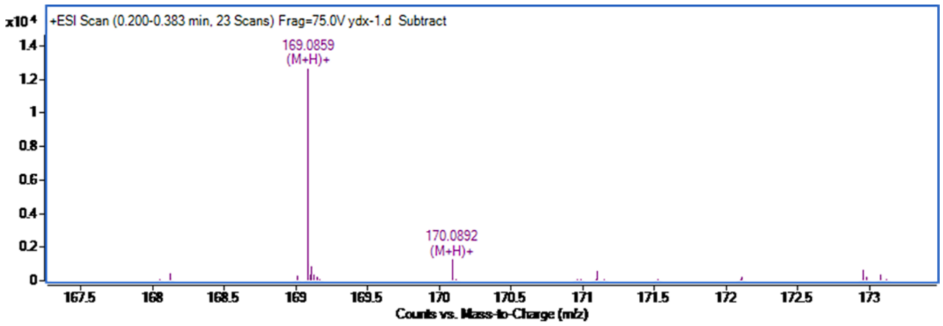

Supplement: Supplementary file 1 [file microorganisms-11-00616-s001.zip › microorganisms-2182563-supplementary.pdf]
